# Divergent Inflammatory Profiles but No Predictive Biomarkers of Psychiatric Sequelae After Viral Infection: A 12-Month Cohort Study

**DOI:** 10.3390/ijms27041670

**Published:** 2026-02-09

**Authors:** Piotr Lorkiewicz, Justyna Adamczuk, Justyna Kryńska, Mateusz Maciejczyk, Małgorzata Żendzian-Piotrowska, Robert Flisiak, Anna Moniuszko-Malinowska, Napoleon Waszkiewicz

**Affiliations:** 1 Department of Psychiatry, Medical University of Bialystok, 15-272 Bialystok, Poland; napwas@wp.pl; 2 Department of Infectious Diseases and Neuroinfection, Medical University of Bialystok, 15-540 Bialystok, Poland; 3 Department of Infectious Diseases and Hepatology, Medical University of Bialystok, 15-569 Bialystok, Poland; 4 Department of Hygiene, Epidemiology and Ergonomics, Medical University of Bialystok, 15-022 Bialystok, Poland

**Keywords:** cytokines, psychiatric disorders, viral infections, SARS-CoV-2, HCV, TBEV, inflammation, biomarkers, neuroimmunology

## Abstract

Viral infections have been implicated in psychiatric outcomes through immune-mediated pathways. This 12-month prospective cohort study, designed as a pilot and hypothesis-generating investigation, compared psychiatric symptoms and inflammatory cytokine profiles in patients with severe acute respiratory syndrome coronavirus 2 (SARS-CoV-2), hepatitis C virus (HCV), and tick-borne encephalitis virus (TBEV), and explored their potential predictive value. Thirty-seven patients hospitalized with viral infections and 32 healthy controls were evaluated, acknowledging the limited sample size. Psychiatric interviews and the Hospital Anxiety and Depression Scale (HADS) were used for assessment. The study was divided into two stages. In Stage 1, during the acute infection, a psychiatric assessment was conducted, and cytokine levels were measured in the patients’ blood. In Stage 2, one year later, the psychiatric assessment was repeated. No significant differences were found in psychiatric diagnosis rates or symptom severity between infection groups, regardless of viral type or neuroinvasive capacity. However, these findings should be interpreted as preliminary given the limited sample size. Some cytokines, eg., interleukin-1β (IL-1β), tumor necrosis factor-alpha (TNF-α), interleukin-10 (IL-10), and soluble interleukin-2 receptor subunit alpha (sIL-2Rα), showed associations with individual symptoms, but these were inconsistent and did not demonstrate robust predictive value. Cluster analysis identified two distinct inflammatory profiles—one characterized by higher cytokine levels (predominantly in Coronavirus disease 2019 (COVID-19) and TBEV cases) and the other by lower cytokine levels (mostly in HCV and controls). However, different cytokine profiles did not correspond to clinical outcomes. The results suggest that psychiatric sequelae after viral infections are not directly driven by specific cytokines or infection type but rather emerge from a complex interaction of immune, psychological, and environmental factors. Single cytokine measurement is insufficient and cannot be used as a tool for assessing the risk of developing psychiatric disorders. Given the exploratory nature of the study, all results require confirmation in larger, adequately powered cohorts. Future studies should focus on composite biomarkers and systems-based models such as neuroimmune-metabolic-oxidative pathways (NIMETOX), or Immune-Inflammatory Response System (IRS)/Compensatory Immune Response System (CIRS)/Oxidative & Nitrosative Stress (O&NS) for improved predictive accuracy.

## 1. Introduction

At the turn of 2019 and 2020, the world faced a pandemic caused by the newly identified severe acute respiratory syndrome coronavirus 2 (SARS-CoV-2). The disease it induces was named coronavirus disease 2019 (COVID-19). The clinical presentation of SARS-CoV-2 infection is highly variable, ranging from asymptomatic and mild cases to severe respiratory failure and even death. Currently, owing to the development of effective vaccines and advances in therapeutic strategies, COVID-19 poses a much smaller threat.

Extensive studies conducted during the pandemic also demonstrated an increased risk of developing psychiatric disorders associated with infection by this virus. Notably, an elevated risk of depression, anxiety, sleep disturbances, manic episodes, and broadly defined psychotic disorders was observed [1]. However, the elevated risk of certain conditions, such as anxiety disorders, appears to be temporary and typically limited to about three months post-infection. In contrast, for others, such as psychotic disorders, the follow-up period remains insufficient, and the risk does not return to baseline levels even up to two years after COVID-19 [2].

Dozens of reports on the association between SARS-CoV-2 and mental health emerging during the pandemic brought renewed attention to the long-known but often overlooked relationship between viral infections and psychiatric disorders.

The impact of viral infections on the development of psychiatric disorders is not a novel concept. Renowned psychiatrists, such as Emil Kraepelin and Karl Menninger, explored this issue as early as the late 19th and early 20th centuries [3,4,5]. Despite the initially promising results, this topic was set aside, and psychiatric research evolved in a different direction.

However, the scale and nature of the COVID-19 pandemic have revived interest in how different viruses affect brain function and psychiatric outcomes. Significant advancements in knowledge and available research methods now make it possible to investigate this topic in ways that were previously unattainable, offering a new perspective and potentially establishing it as a meaningful etiopathogenic factor in mental disorders.

Studies indicate that various viruses can alter nervous system function through mechanisms such as neurotropism, chronic inflammation, oxidative stress, neurotransmitter disturbances, virus–host protein–protein interactions and microbiota dysbiosis [6,7,8,9,10,11].

Viruses with different modes of action, such as SARS-CoV-2, hepatitis C virus (HCV), and tick-borne encephalitis virus (TBEV), may affect the central nervous system (CNS) in distinct ways. Because of that, analyzing their impact could enhance our understanding of the etiology of psychiatric disorders.

The mechanisms leading to psychiatric disturbances may include neurotropism and neuroinvasiveness, as some viruses can directly infect CNS cells or cross the blood–brain barrier (BBB). Of the viruses analyzed in this study, TBEV has confirmed neurotropic properties [12], while SARS-CoV-2 exhibits partial neurotropism [13,14] as well as neuroinvasive capabilities [9]. Evidence of HCV neuroinvasiveness remains limited [15,16]. Viral infections may also trigger immune activation with cytokine dysregulation, leading to neurovirulence. Excessive immune responses with elevated cytokine levels, e.g., interleukin-1β (IL-1β), interleukin-6 (IL-6), interleukin-8 (IL-8), interleukin-10 (IL-10), tumor necrosis factor-alpha (TNF-α), and interferons have been reported [6]. Such immune activation may disrupt neurotransmitter function and contribute to psychiatric symptoms [17]. These cytokines may also compromise the BBB and promote neuroinflammation. Viruses such as SARS-CoV-2 and HCV additionally can induce immune dysregulation, oxidative stress, hypoxia, prothrombotic abilities and metabolic disturbances. Together, these mechanisms may cause CNS pathology without direct viral invasion, which is referred to as neurovirulence [9,15,16,18,19,20]. Chronic viral-induced stress may also lead to dysregulation of the hypothalamic–pituitary–adrenal (HPA) axis, resulting in increased cortisol levels and heightened susceptibility to anxiety, depression, and psychosis [21,22,23]. Increased levels of inflammatory cytokines and viral components can cause neurotransmitter imbalance and alter serotonin (5-HT), dopamine (DA), and glutamate (Glu) pathways [17,24]. Viral infections also activate the kynurenine pathway, resulting in increased neurotoxic metabolites such as quinolinic acid, which is linked to excitotoxicity and mood disturbances [25,26]. Another relevant mechanism involves virus–host protein–protein interactions (PPIs). Through interactions between viral and host proteins, viral infections can modulate host biological processes and disrupt critical systems, including immune regulation. PPIs also influence β-amyloid accumulation, reactive oxygen species production, neuronal death, gliogenesis, and autophagy. This type of dysregulation may contribute to the development of complex diseases, including neurological and neuropsychiatric disorders [11,27]. Finally, microbiota dysbiosis may play an important role. The bidirectional cross-talk between commensal microbiota and the CNS, known as the gut–brain axis, plays a crucial role in regulating immune, neuroendocrine and inflammatory pathways. Through the activity of the gut microbiota, neurotransmitters such as Glu, γ-aminobutyric acid (GABA), DA, norepinephrine (NE), and 5-HT are produced [28]. Dysbiosis and gastrointestinal inflammation, accompanied by increased intestinal and BBB permeability, allow cytokines and neurotransmitters released in the gut to enter the CNS, leading to its dysfunction and increasing the risk of psychiatric disorders such as depression and anxiety [10,29].

In continuity with our previous publications, this study focuses on the impact of virus-induced inflammation and cytokine dysregulation on the development of psychiatric disorders, as immunological alterations underlie other pathogenic mechanisms and constitute their common link [6,25,30]. It aims to assess the prevalence of psychiatric symptoms and disorders following infection with SARS-CoV-2, HCV, and TBEV, and to examine associated inflammatory biomarkers.

Based on previous evidence, it was hypothesized that infection with SARS-CoV-2 would be associated with a higher risk of developing mental disorders compared to HCV and TBEV. This hypothesis was formulated based on reports on 6-month neurological and psychiatric outcomes in patients with SARS-CoV-2 infection, among whom 24% were diagnosed with a mood, anxiety, or psychotic disorder (with 8.6% being first-ever diagnoses) [31]. By contrast, among patients after tick-borne encephalitis, only 1.5% received a psychiatric diagnosis one month after discharge [32]. In the case of HCV, some studies indicate that more than 30% of patients exhibit mental disorders (depression and anxiety) prior to antiviral treatment [33]. We also considered the inflammatory theory of mental disorders [34,35,36] and the fact that SARS-CoV-2 elicits a cytokine storm that may substantially contribute to the development of psychiatric disorders, potentially translating into a higher risk compared with other viral infections [37,38]. It was further hypothesized that each virus induces a distinct cytokine profile, potentially serving as a biomarker for psychiatric outcomes. This hypothesis arose from our previous observations and studies, in which we noted that the cytokine dysregulation profile we established across multiple psychiatric conditions, although generally similar, often differs by changes in one or more cytokines. A comparable pattern is seen in viral diseases [6]. Variations in cytokine levels have also been reported by other authors [39]. Given the subtle and complex influence of the immune system on psychiatric disorders, such small differences could, in theory, determine disease development. These differences, however, required testing under conditions identical for every participant. The third and final hypothesis assumed that the degree of CNS involvement during infection varies depending on the type of virus (neurotropism, neuroinvasiveness, neurovirulence) and may influence psychiatric outcomes, but is not their primary determinant. This hypothesis is also grounded in our earlier observation that some viruses not generally considered neurotropic (e.g., Human Immunodeficiency Virus—HIV, or H1N1 influenza) are more frequently linked to psychiatric complications than certain viruses with strong neurotropism, such as Epstein–Barr Virus (EBV) [6]. This would suggest that affinity for neural cells may not be as important factor as it might seem.

Given the methodological complexity of comparative viral studies and the limited sample size, the present investigation was designed as a pilot, hypothesis-generating study, and all findings should be interpreted as preliminary.

Within this exploratory framework, the findings of this study may contribute to a better understanding of the pathogenesis of mental disorders and help inform the development of future diagnostic and therapeutic strategies. Moreover, the methodological and data-analytic findings of this study highlight the need for pilot research prior to drawing definitive conclusions.

## 2. Results

### 2.1. Group Characteristics

No significant differences were found between groups in sex, vaccination status, or psychiatric history. However, participants with TBEV were significantly younger than controls and COVID-19 patients. Tobacco use was highest in the HCV group. COVID-19 patients more frequently reported heart disease and cancer, while hyperlipidemia was more common in controls [Tables S1–S4].

### 2.2. Descriptive Statistics for Quantitative Variables

Descriptive statistics for psychiatric outcomes (HADS scores) and plasma biomarkers were calculated at both the aggregated level and stratified by viral infection type. Most variables deviated from normal distribution, though skewness was minor for the majority. Parametric tests were applied, except for highly skewed biomarkers such as interferon alpha-2 (IFN-α2), interleukin-2 (IL-2), IL-6, IL-8, and interleukin-12 subunit p40 (IL-12(p40)), for which non-parametric tests were used [Tables S5–S6A].

### 2.3. Comparison of Psychiatric Symptoms

No significant differences in psychiatric symptom prevalence were found across patient groups at either time point [Tables S7 and S8]. Additionally, no differences were observed between the groups in psychiatric symptom changes over the course of the study [Table S9].

**Figure 1:**
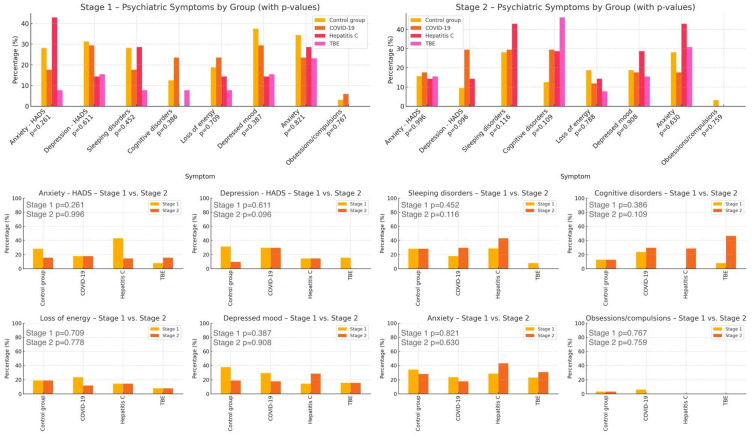
Comparison of Psychiatric Symptoms by Diagnosis—Stage 1 and Stage 2: COVID-19—coronavirus disease 2019, HADS—Hospital Anxiety and Depression Scale, TBE—tick-borne encephalitis, p—statistical significance, stage 1—baseline, stage 2—after 12 months.

These findings suggest that one year after infection with a given virus, patients are not at greater risk of developing or changing the severity of the assessed psychiatric symptoms compared to individuals in the control group. A visual representation of these results is provided in Figure 1.

**Figure 2:**
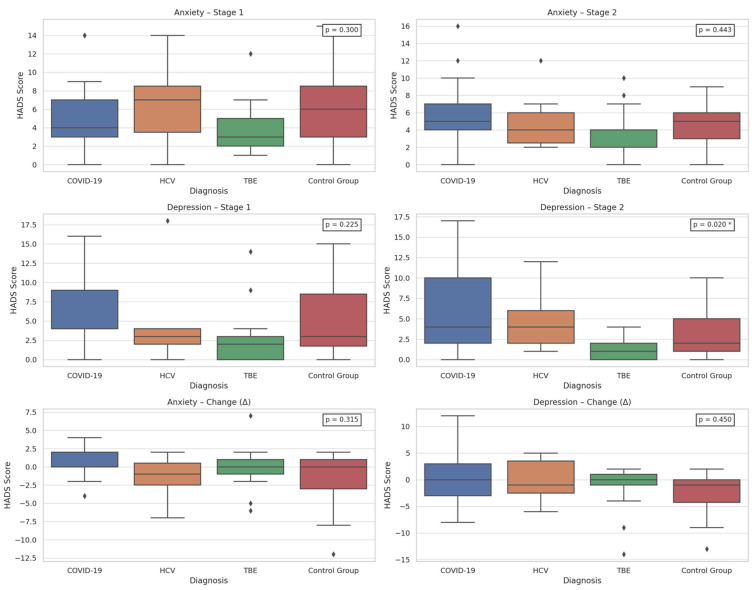
Comparison of HADS score by group—Stage 1 and Stage 2: COVID-19—coronavirus disease 2019, HADS—Hospital Anxiety and Depression Scale, HCV—Hepatitis C virus, TBE—tick-borne encephalitis, *p*—statistical significance, stage 1—baseline, stage 2—after 12 months, *—statistical significance.

Additionally, the type of viral infection was significantly associated only with depression severity at Stage 2, as assessed by the HADS (*p* = 0.020). Post hoc analysis showed higher depression levels in COVID-19 patients compared to those with TBEV (*p* = 0.022) [Tables S10–S12]. Graphical representation is provided in Figure 2. To further clarify this result, Table 1 presents the full post hoc Dunn test results.

### 2.4. Comparison of Psychiatric Diagnoses

**Figure 3:**
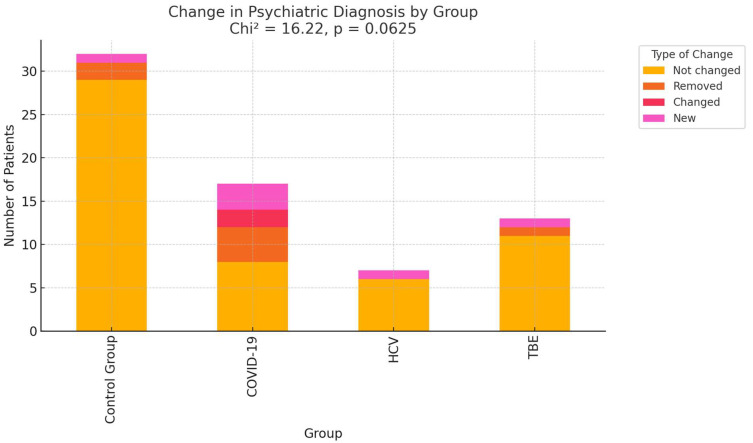
Comparison of change in psychiatric diagnosis between stages 1 and 2 by group: COVID-19—coronavirus disease 2019, HCV—Hepatitis C virus, *p*—statistical significance, TBE—tick-borne encephalitis.

Adjustment disorder, depressive episodes, and mixed anxiety-depression were most common in both stages of the study [Tables S13 and S14]. No statistically significant differences in diagnosis progression were observed across infection groups [Table S15]. The results are illustrated graphically in Figure 3.

However, a non-significant trend suggested more frequent changes in psychiatric diagnoses in the COVID-19 group (*p* = 0.0625, Cramér’s V = 0.28). This trend may indicate that COVID-19 infection is associated with greater variability or progression in mental health status.

The type of psychoactive substances used by patients did not significantly influence the likelihood of a diagnostic change [Table S16]. However, patients with comorbid cancer or asthma/chronic obstructive pulmonary disease (COPD) were significantly more likely to experience diagnostic changes [Table S17].

### 2.5. Comparison of Cytokine Dysregulation and Their Associations to Psychiatric Symptoms

After excluding a direct link between virus type and psychiatric disorders, the analysis focused on associations between viral infection and cytokine levels measured at Stage 1, as well as their relation to psychiatric outcomes. To account for multiple testing, false discovery rate (FDR) correction using the Benjamini–Hochberg procedure was applied. The results are presented in Table 2.

**Figure 4:**
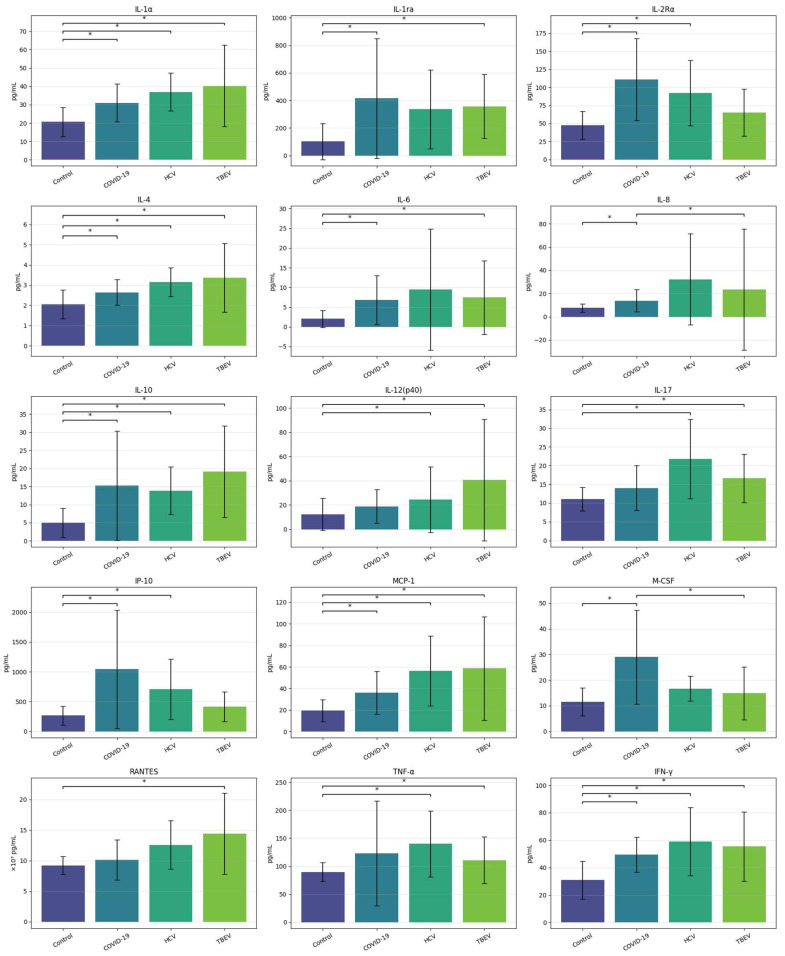
Cytokine Levels—Annotated Comparisons Between Groups. This figure summarizes the mean serum levels of 15 cytokines across four diagnostic groups: Control, COVID-19, HCV, and TBEV. Bars represent mean values; vertical error bars indicate standard deviation (SD). Statistically significant differences (*p* < 0.05) between group pairs are marked with an asterisk (*) above the corresponding bars. Each horizontal line connects two groups that showed a significant difference according to Dunn’s post hoc test following the Kruskal–Wallis test. Abbreviations: IFN-γ—interferon gamma; IL-1α—interleukin 1 alpha; IL-1ra—interleukin-1 receptor antagonist; IL-2Rα—interleukin-2 receptor alpha (soluble alpha subunit); IL-4—interleukin 4; IL-6—interleukin 6; IL-8—interleukin 8; IL-10—interleukin 10; IL-12(p40)—interleukin 12, p40 heterodimer; IL-17—interleukin 17; IP-10—interferon gamma-induced protein 10; MCP-1—monocyte chemoattractant protein 1; M-CSF—macrophage colony-stimulating factor; RANTES—regulated on activation, normal T cell expressed and secreted; TNF-α—tumor necrosis factor alpha.

The analysis revealed statistically significant differences between groups in the levels of IFN-α2, IFN-γ, interleukin-1 alpha (IL-1α), interleukin-1 receptor antagonist (IL-1ra), interleukin-2 receptor alpha (IL-2Rα), interleukin-4 (IL-4), IL-6, interleukin-7 (IL-7), IL-8, IL-10, IL-12(p40), interleukin-17 (IL-17), Interferon gamma-induced protein 10 (IP-10), Monocyte Chemoattractant Protein-1 (MCP-1), Macrophage Colony-Stimulating Factor (M-CSF), Regulated upon Activation, Normal T Cell Expressed and Secreted (RANTES), and TNF-α. Controls generally showed lower levels than infected groups, particularly those with COVID-19 and TBEV. No statistically significant differences were observed for IFN-α2 and IL-7 after correction for multiple comparisons. Full results are presented in [Tables S18A–S18F]. Graphically they are illustrated in Figure 4.

Based on the statistically significant differences in cytokine levels, dominant cytokine profiles associated with each viral diagnosis were constructed and are summarized in Table 3.

Next, cytokines predicting psychiatric diagnoses and symptoms were identified. To this end, hierarchical logistic regression models were constructed. Due to multicollinearity, variables with a variance inflation factor (VIF) > 5.00 were excluded from further analyses. Furthermore, given the large number of predictors relative to the sample size, the analysis employed backward elimination to obtain a best-fitting model while excluding variables with low predictive power.

In Stage 1 of the study, the significant predictors of psychiatric diagnosis included IFN-α2, IL-2Rα, IL-17, and IP-10. In Stage 1, higher levels of IFN-α2, IL-17, and IP-10 were linked to reduced risk of diagnosis, by 70%, 21%, and less than 1% respectively, while IL-2Rα was associated with 6% increase in risk of diagnosis. In Stage 2, only RANTES predicted a slight increase in risk—by less than 1%. Cytokines significantly predicting specific symptoms across stages are summarized in [Table S19].

With regard to specific symptoms, Table 4 lists cytokines that significantly predicted symptom probability at both stages. Detailed statistics are in [Tables S20 and S21].

Only a few cytokines, such as IL-12(p40), IL-2Rα, and RANTES, were significant predictors at both stages. Others, like IL-2, SCGF-β, IL-8, MIP-1α, IL-1β, and TNF-α were predictive only at Stage 2. Cytokines like IL-7, IL-9, IL-17, IP-10, and IL-10 were predictive at Stage 1 but lost significance over time.

An additional analysis examined cytokine effects on anxiety and depression severity (HADS, continuous). Hierarchical logistic regression models were used again. Predictors with a VIF > 5 were excluded to mitigate multicollinearity, and backward elimination was applied to obtain the best-fitting model. After excluding highly collinear markers, IL-1β predicted anxiety at Stage 1 (weak effect). Depression severity was linked to IL-10 (positive) and RANTES (negative) at Stage 1. At Stage 2, depression severity was positively linked to increasing concentrations of IP-10 and SCGF-β. Full data are presented in [Tables S22 and S23].

**Figure 5:**
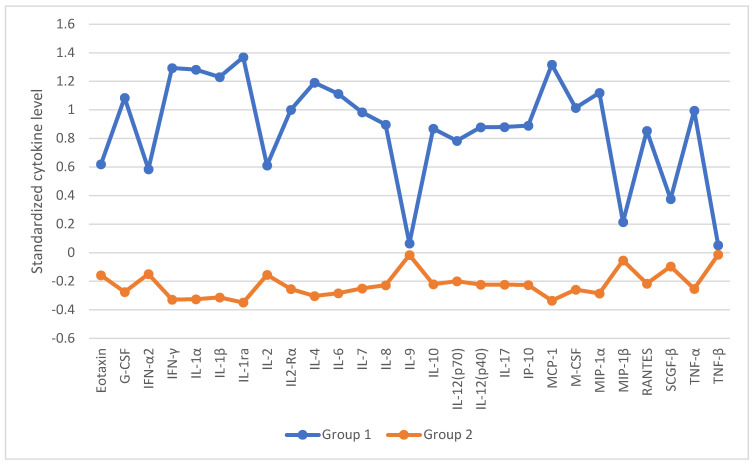
Standardized mean values of cytokine levels depending on the identified group. Abbreviations: G-CSF—granulocyte colony-stimulating factor; IFN-α2—interferon alpha 2; IFN-γ—interferon gamma; IL-1α—interleukin 1 alpha; IL-1β—interleukin 1 beta; IL-1ra—interleukin 1 receptor antagonist; IL-2—interleukin 2; IL-2Rα—interleukin 2 receptor alpha (soluble alpha subunit); IL-4—interleukin 4; IL-6—interleukin 6; IL-7—interleukin 7; IL-8—interleukin 8; IL-9—interleukin 9; IL-10—interleukin 10; IL-12(p70)—interleukin 12, p70 heterodimer; IL-12(p40)—interleukin 12, p40 subunit; IL-17—interleukin 17; IP-10—interferon gamma-induced protein 10; MCP-1—monocyte chemoattractant protein 1; M-CSF—macrophage colony-stimulating factor; MIP-1α—macrophage inflammatory protein 1 alpha; MIP-1β—macrophage inflammatory protein 1 beta; RANTES—regulated upon activation, normal T cell expressed and secreted; SCGF-β—stem cell growth factor beta; TNF-α—tumor necrosis factor alpha; TNF-β—tumor necrosis factor beta.

In the final analysis of cytokine dysregulations, patients were clustered by cytokine profiles using two-step cluster analysis. In result, two distinct clusters emerged, differing significantly in most cytokine levels. Cluster 1 (*n* = 14) showed higher cytokine levels than Cluster 2 (*n* = 55). The cytokine profiles of the identified clusters are illustrated in Figure 5. Higher cytokine profiles were linked to COVID-19 or TBEV, while lower profiles were more common in controls and HCV cases.

No significant cluster differences were found in sleep, cognition, energy, anxiety, or depression symptoms at either stage of the study. Similarly, HADS-measured anxiety and depression severity did not differ between clusters. Full results are presented in [Tables S24–S26].

### 2.6. Impact of Prior COVID-19 Infection on the Course of Psychiatric Symptoms and Diagnoses

The final analysis assessed whether prior COVID-19 infection affected psychiatric outcomes. No significant group differences were found in diagnostic changes, symptom presence, or HADS scores at either stage. However, those with a COVID-19 history showed a significantly greater reduction in depression severity over time. Results in [Tables S27–S33].

## 3. Discussion

Given the methodological complexity of comparative studies and the multiple sources of clinical and immunological heterogeneity, pilot studies play a crucial role in generating testable hypotheses and informing the design of adequately powered confirmatory research. This study aimed to compare the prevalence of psychiatric disorders after SARS-CoV-2, HCV, and TBEV infections and identify related cytokine biomarkers. It was hypothesized that SARS-CoV-2 posed the greatest psychiatric risk among analyzed viruses and that their distinct cytokine profiles might predict outcomes. Additionally, it was assumed that the neuroinfectious properties of the studied viruses differ, and that these differences influence the frequency and severity of neuropsychiatric manifestations; however, these properties are unlikely to be their primary determinant. Importantly, given the exploratory and pilot nature of the present study, all hypotheses were tested in a hypothesis-generating framework rather than to establish definitive causal relationships.

Demographic data showed TBEV patients were significantly younger, likely due to greater tick exposure in active individuals, while COVID-19 patients more often had cardiovascular disease and cancer—aligning with known risk patterns in older, comorbid populations [40,41]. These baseline differences should be taken into account when interpreting the findings, particularly in the context of the limited sample size.

Unexpectedly, no significant group differences in psychiatric symptom frequency were found at either study stage. The only exception was greater depressive symptom severity after COVID-19 compared to TBEV in Stage 2. This may reflect the more acute and self-limiting nature of TBEV—potentially promoting a faster neuroimmune return to homeostasis [42], its lower psychosocial burden, and the younger, healthier profile of TBEV patients. In contrast, COVID-19 is associated with prolonged, systemic inflammation as well as substantial psychological stress and health-related anxiety [25,43].

No significant group differences were found in diagnostic changes over one year, though COVID-19 patients showed a trend toward greater mental health variability. Early post-COVID-19 symptoms often appear within three months, after which the risk tends to return to population baseline levels. In those without improvement by six months, symptoms may recur or worsen even 2–3 years post-COVID-19, regardless of initial illness severity [2,44]. This pattern of early symptom development may ultimately influence the clinical presentation and contribute to diagnostic changes observed at the one-year mark. However, cancer, more common in the COVID-19 group, was linked to greater risk of diagnostic change which also may affect observed variability of mental health in COVID-19 group. These trends remain preliminary and require confirmation in larger, adequately powered cohorts.

Analysis of all collected data indicates that the type of virus, regardless of its neuroinfectious properties, did not significantly determine the frequency of psychiatric symptoms or diagnoses. Consequently, the first research hypothesis was not supported, whereas the third research hypothesis was sustained. Given the pilot design of the study, these conclusions should be regarded as provisional.

Despite marked biological differences between the analyzed viruses, these differences did not translate into distinct psychiatric outcomes. SARS-CoV-2, HCV, and TBEV vary greatly in their mode of transmission, course of infection, neurotropic properties, and patterns of immune activation. Within the exploratory framework of this study, viral characteristics appeared insufficient to explain psychiatric outcomes on their own.

SARS-CoV-2, an enveloped, positive-sense single-stranded RNA virus belonging to the *Coronaviridae* family, predominantly causes an acute type of infection, and transmission occurs mainly through respiratory droplets and aerosols. The infection primarily affects the respiratory system and in severe cases may lead to viral pneumonia and acute respiratory distress syndrome (ARDS). It can also directly affect other organ systems, including the circulatory, gastrointestinal, excretory, endocrine, and nervous systems [45,46]. SARS-CoV-2 shows tropism for pulmonary epithelial and endothelial cells. It is also believed to exhibit neuroinvasiveness and affinity for glial cells, while its neurotropism has not been conclusively confirmed in human studies [13,14].

HCV is also an enveloped, positive-sense single-stranded RNA virus, classified within the Flaviviridae family. In contrast to SARS-CoV-2, it usually causes a chronic infection that follows an acute phase. Chronic infection is often asymptomatic or mild. Transmission occurs via the parenteral route through infected blood. This virus infects hepatocytes and leads to chronic inflammation, fibrosis, cirrhosis, and hepatocellular carcinoma. However, it is not directly hepatotoxic and most of the liver damage results from a cell-mediated immune reaction against infected liver cells. HCV may also cause extrahepatic manifestations, including metabolic, cardiovascular, autoimmune, renal, thyroid, and pulmonary disorders [47]. Neuropsychiatric symptoms may include depression and cognitive impairment. The neuroinvasiveness of HCV remains a matter of debate, although some reports suggest that it may replicate within brain microvascular endothelial cells, astrocytes, and microglia [15,16].

TBEV, similarly to the previous virus, is an enveloped, positive-sense single-stranded RNA virus belonging to the Flaviviridae family. It usually causes acute infection and is transmitted through the bite of infected ticks. The clinical course often begins with flu-like symptoms, followed by a short remission period. After that, the second phase develops and it is characterized by meningitis, encephalitis, or meningoencephalomyelitis. Symptoms of meningitis are present in most patients and include high fever, headache, nausea, and vomiting. Encephalitic manifestations may involve impaired consciousness, personality changes, behavioral disturbances, cognitive dysfunction and tremor of the extremities. In some cases, delirium and psychosis may also occur. Meningoencephalomyelitic forms are characterized by flaccid pareses. Unlike previous two, TBEV displays marked, undeniable neurotropism, infecting neurons and glial cells, which may result in lasting neurological and psychiatric sequelae [12,48].

The absence of differences in the prevalence or trajectory of psychiatric symptoms observed in this cohort, despite obvious differences in viral characteristics, may suggests that the decisive factor may lie not in the intrinsic properties of the virus itself, but rather in its impact on the host’s immune response.

This raises a legitimate question: could the intensity of the inflammatory response, regardless of the specific pathogen, be the key factor contributing to the development of psychiatric disorders? Although the control group exhibited the lowest levels of inflammatory markers, it did not show a lower frequency of psychiatric symptoms compared to the infected groups. This finding challenges the notion of a straightforward relationship between inflammation severity and psychiatric risk. Nevertheless, the limited sample size constrains the ability to draw firm conclusions regarding these relationships.

Regarding the second research hypothesis: each virus showed a distinct cytokine profile—COVID-19 with a systemic cytokine storm, HCV with chronic Th1/Th17 activation, and TBEV with neuroinflammation involving CNS chemotaxis (e.g., RANTES).

Observed cytokine alterations may be related to virus-specific immune mechanisms and interactions with particular viral proteins, rather than representing a nonspecific inflammatory response. For example, it has been observed that in SARS-CoV-2 infection, activation of monocytes and macrophages plays a central role in initiating and sustaining the cytokine storm [49]. At the level of soluble mediators, this response is commonly reflected by increased levels of pro-inflammatory cytokines such as IL-1β, TNF-α, and IL-6, as well as chemokines involved in effector cell recruitment, including IP-10 and IL-8 [50]. Importantly, it has been demonstrated that the S1 subunit of the SARS-CoV-2 spike protein can directly activate Toll-like receptors 4 (TLR4) on macrophages, triggering the nuclear factor kappa B (NF-κB) signaling pathway and promoting the release of inflammatory cytokines [51]. In contrast, HCV infection is typically associated with a state of chronic antigen-driven immune stimulation, leading to stabilization of cytokine patterns and persistent expression of markers indicative of lymphocyte activation. In this context, the Th1/Th17 axis, including IL-17, is prominently involved, and this type of immune response has been shown to correlate with the severity of inflammation and fibrosis [52]. In addition, infected cells, particularly hepatocytes, release mediators that promote activation of dendritic cells and monocytes toward Th17 differentiation [53]. During the acute phase of infection, type I and type III interferons play a major role, while virus-specific antibodies are primarily directed against the envelope glycoproteins E1 and E2 [54]. In the case of TBEV, which exhibits clear neurotropism, mediators facilitating leukocyte trafficking into the central nervous system appear to be particularly relevant. Elevated levels of IP-10 have been reported both in serum and, more importantly, in cerebrospinal fluid, consistent with active recruitment of immune cells into the CNS [55,56]. TBEV has also been shown to directly induce RANTES, with the viral NS5 protein implicated in this process [57,58].

However, no cytokine profile observed it this study was consistently linked to specific psychiatric symptoms, contradicting, for example, the inflammatory theory of depression, which posits that activation of Th1 and Th17 axes correlates with increased affective symptom severity [34], as well as other prior findings on cytokine elevation in psychiatric disorders [6].

This suggests inflammation alone may not cause psychiatric disorders; additional factors, e.g., genetic vulnerability, trauma, stress, substance use or personality disorders, may be needed for symptoms to emerge. Both biological and environmental risks likely play a role in translation of inflammatory response to clinically relevant psychiatric symptoms [59,60]. Importantly, chemotactic markers associated with immune cell migration into the CNS may be more sensitively detected in cerebrospinal fluid than in peripheral blood. Therefore, the lack of a clear correlation between peripheral cytokine profiles and psychiatric symptoms does not necessarily negate a role for immune-mediated mechanisms in the development of these symptoms but may instead reflect the limitations of relying on peripheral measurements to capture localized neuroinflammatory processes.

Despite differences in the dominant cytokines i.e., ‘cytokine profiles’ observed across various viral infections, these differences did not influence the presence or absence of specific psychiatric diagnoses among infected patients. Therefore, we were unable to confirm our second research hypothesis. Nevertheless, certain cytokines, when considered independently of the type of viral infection and psychiatric diagnosis, were associated with specific psychiatric symptoms. However, as cytokines were measured only once during the study, this limits conclusions about immune dynamics and its influence on psychiatric disorders. Despite aiming to test the predictive value of a single measurement, this remains a key study limitation requiring future longitudinal research.

The following section discusses the observed associations of cytokines and psychiatric symptoms by cytokine function—from pro-inflammatory Th1/Th17 mediators to immunoregulatory factors and CNS-directed chemokines.

### 3.1. Th1 Pro-Inflammatory Cytokines

IL-1β is a potent pro-inflammatory cytokine whose excess has been linked to impaired synaptic plasticity, reduced neurogenesis and depressive symptoms. It disrupts glutamatergic homeostasis, enhances excitotoxicity, and may promote oxidative stress. Numerous studies indicate that elevated IL-1β levels correlate with reduced hippocampal volume, executive function deficits, and increased vulnerability to neurodegeneration [61,62,63]. In Stage 2 of our study, IL-1β predicted nearly double the risk of cognitive impairment. Highest levels, although statistically insignificant, were seen in the HCV group, which also had more cognitive deficits. The results support previous findings, that higher IL-1β levels during acute infection may raise the risk of long-term cognitive impairment, though environmental influences cannot be excluded. The lack of strong effect between groups may stem from serum IL-1β not reflecting CNS levels or its effects being modulated by regulatory cytokines like IL-10 or IL-1ra.

TNF-α is a key pro-inflammatory cytokine whose effects depend on signaling through two receptors: TNFR1, associated with inflammation and neuronal damage; and TNFR2, which may exert neuroprotective effects and limit microglial toxicity. TNF-α effects depend on receptor expression and activation [64,65,66,67]. While chronically elevated TNF-α has been linked to synaptic dysregulation, microglial activation, and cognitive decline, its transient increases during acute inflammation may play a protective role by stabilizing the BBB and modulating excitotoxicity [66,67]. In our study, TNF-α levels were significantly higher in the TBEV and HCV groups, which also showed the largest increase in cognitive impairments. Paradoxically, higher TNF-α levels at Stage 1 were associated with a 10% reduction in the risk of cognitive impairment at Stage 2, possibly indicating early TNFR2-mediated neuroprotection. Groups with the highest TNF-α initially had the fewest deficits, suggesting a well-regulated inflammatory response. A decline in TNF-α over time may have allowed other cytokines, like IL-8 or IL-1β, to exert greater effects on cognition.

### 3.2. Th17/Neuroinflammatory Cytokines with an Autoimmune Component

IL-17 showed a protective effect in the first stage of the study. Its increase was linked to a 76% lower risk of cognitive impairment and 12% less anxiety. Highest IL-17 levels were found in TBEV and HCV patients, correlating with fewer cognitive deficits. While typically associated with CNS damage [68,69], emerging evidence suggests IL-17 may have neuroprotective effects, possibly depending on its concentration and immune context. During infection, it may limit neuronal apoptosis and exert the protective effects observed in our cohort, suggesting that the dominant function of IL-17 depends on the phase and nature of the immune response [70].

IL-12(p40) plays a modulatory role within the Th1/Th17 immune axis, acting both as part of the pro-inflammatory IL-12(p70) heterodimer and, in its homodimeric form (p40)_2_, as an immunoregulatory factor that can inhibit Th1 and Th17 signaling [71,72]. In our study, its levels correlated with greater depressive and anxiety symptoms at both study stages. However, the highest concentrations were observed in the HCV and TBEV groups, where affective symptoms were less prevalent than in COVID-19 patients. This may indicate a dominance of the (p40)_2_ homodimer, which blocks Th1/Th17 signaling. In COVID-19, IL-12(p40) may have served a compensatory but insufficient role. Its dual function, as proinflammatory in the IL-12(p70) heterodimer and immunoregulatory in the (p40)_2_ form, could explain observed inconsistencies. The shift from anxiety-related effects in Stage 1 to depression in Stage 2 may reflect symptom evolution from acute stress to chronic mood disturbances.

### 3.3. Anti-Inflammatory/Immunoregulatory Cytokines

IL-10 is an anti-inflammatory cytokine that modulates immune responses and limits tissue damage [73]. Although often decreased in depression and anxiety, elevated IL-10 has been noted in psychosis and may reflect a compensatory anti-inflammatory response [6,17,74]. In our study, higher IL-10 levels in Stage 1 were linked to reduced energy. which may be consistent with activation of the CARS (Compensatory Anti-inflammatory Response Syndrome), a protective, anti-inflammatory response to strong immune system activation that can nonetheless contribute to fatigue and low mood [75]. In Stage 2, higher IL-10 levels during acute infection predicted a lower risk of later sleep disturbances, suggesting a neuroprotective role in shielding sleep-regulating brain regions from proinflammatory cytokine damage [73,76].

SCGF-β is produced by bone marrow cells and supports hematopoiesis, tissue regeneration, and immune regulation. Though not significantly elevated in any group, its increase was linked to a 1% reduction in depression risk. This may reflect its ability to support immune system regeneration, suppress neuroinflammatory mechanisms within the CNS, and enhance neuroimmune balance and resilience to stressors. Lower SCGF-β levels have also been observed in the cerebrospinal fluid (CSF) of Systemic Lupus Erythematosus (SLE) patients with depression [77].

### 3.4. Receptors and Markers of Lymphocyte Activation

sIL-2Rα is a soluble form of the alpha subunit of the interleukin-2 receptor, and marker of T-cell activation and systemic immune response. Compared to control group, sIL-2Rα was significantly elevated in the COVID-19 and HCV groups and were associated with an increased risk of cognitive impairment at both study stages [78,79,80]. While group differences were not statistically significant, the COVID-19 group showed nearly double the impairment rate early on. In HCV and TBEV, rates increased over time. The absence of clear group-level differences may reflect limited statistical power and modulation by immunoregulatory factors (e.g., IL-1ra, IL-10 which may have mitigated the neuroinflammatory impact of sIL-2Rα or demographic differences (age, comorbidities).

IL-2 supports T-cell and NK cell activity. However, at low levels, it promotes Treg development, helping suppress inflammation and maintain immune balance, potentially protecting the CNS against prolonged post-viral inflammation [81,82]. In our study, higher IL-2 levels predicted a lower risk of depression in Stage 2. Although not significantly elevated across groups, moderate IL-2 level increase may have immunoregulatory effects. In line with our findings, low-dose IL-2 is also being investigated experimentally as a treatment for depression and post-traumatic stress disorder (PTSD) [83,84].

IL-7 supports T and B cell survival and proliferation, and is elevated in immune dysregulation, such as HIV [85,86]. Though typically reduced in depression, excessive IL-7 activity may sustain chronic inflammatory responses and has been previously linked to high-risk schizophrenia, correlating with the severity of positive symptoms [87,88]. In our study it was linked to a 12% higher risk of depression in Stage 1. Group differences were initially significant but lost significance after correction. IL-7’s predictive value was limited over time. According to literature, its association with depressive symptoms may vary by sex and blood fraction, with opposing trends in men and women [89].

### 3.5. Chemokines—Cell Migration to the CNS

RANTES is a chemokine secreted by activated T cells. It recruits immune cells to inflammation sites and activates microglia and astrocytes in the CNS [90]. In Stage 1, its increase was linked to reduced risk of low mood and energy loss but higher risk of cognitive impairment. In Stage 2, higher RANTES predicted a 1% increase in anxiety and cognitive disorders. RANTES levels were highest in the TBEV group, though depressive symptoms were more frequent in controls. Based on these results it is possible that RANTES has a biphasic effect, meaning that initially it may be protective, but its chronic elevation may promote low-grade CNS inflammation and prolonged immune activation, possibly leading to neurotoxicity, persistent cognitive deficits, and heightened anxiety. These results align with some of the previous findings in patients with Alzheimer’s disease and ischemic stroke [91,92,93].

MIP-1α is a proinflammatory CC chemokine produced by various immune and glial cells. It modulates inflammation and acts as a chemoattractant. In our study it was linked to a 74% reduced risk of cognitive impairment in Stage 2, despite no significant group differences in its levels. Our findings stand opposite to previous studies which associate MIP-1α with cognitive decline [94,95], possibly due to differences in immune activation phase (acute vs. resolving), clinical context, or characteristics of the study population. In a resolving inflammatory state, MIP-1α may support neuroprotection through microglial clearance of damaged structures, promoting neuronal pathway reorganization, or contributing to the regulation of the local neuroimmune environment [96].

IP-10 (CXCL10) is induced by IFN-γ, TNF-α, and IL-1β, and it recruits Th1 cells and NK cells. In the brain, it is produced by astrocytes and microglia [97]. In our research, higher IP-10 levels in Stage 1 were associated with a reduced risk of depressive symptoms. Although IP-10 concentrations were higher in the COVID-19 and HCV groups, depressive symptoms were not more severe than in controls, who paradoxically showed more depressive features despite lower IP-10 levels. Previous findings regarding IP-10 and depression are inconsistent [97,98]. In this cohort, IP-10 may reflect a well-regulated Th1 response or exert a transient neuroprotective effect during infection, while the single time-point measurement likely failed to capture the full dynamics of immune activation. Moreover, some studies suggest a potential neuroprotective role of IP-10 during infection, which in the context of pathogen-induced psychiatric symptoms, could help reduce the risk of depression [99].

IL-8 is a pro-inflammatory chemoattractant produced by microglia, astrocytes, and endothelial cells. It facilitates the migration of immune cells across the BBB [100]. In our study, higher IL-8 levels were associated with an increased risk of cognitive impairment at follow-up, consistent with previous reports [100,101,102]. Although IL-8 concentrations were highest in the COVID-19 group and lowest in TBEV and controls, cognitive decline was most pronounced in TBEV, possibly reflecting direct neurotropic effects and CNS injury rather than peripheral cytokine activity. Previous studies have reported elevated IL-8 levels in TBEV, particularly in CSF, while serum levels may not fully reflect localized neuroinflammation [103]. Our findings suggest that cognitive impairment may result not only from cytokine activity but also from direct CNS injury and other mechanisms. Notably, one randomized clinical trial found that higher IL-8 levels might have protective effects against depression in low-dose endotoxin studies, especially in women [104,105]. Similarly, in our cohort, the lowest IL-8 levels were linked to the greatest cognitive decline.

### 3.6. Cluster Analysis

The conducted cluster analysis identified two distinct inflammatory profiles: one comprising 14 individuals, characterized by elevated cytokine levels, and another, larger cluster of 55 individuals with low cytokine concentrations. Patients in the high-inflammation cluster were more likely to have had COVID-19 or TBEV infections, suggesting that these pathogens may have the greatest potential to induce substantial immunological disturbances. However, not all patients in COVID-19 or TBEV groups exhibited markedly elevated cytokine levels.

No significant differences were found between clusters in psychiatric diagnoses or symptom severity, suggesting that a globally elevated inflammatory profile does not directly translate into clinical symptoms. Individual cytokine effects, which were found significant in regression analyses, may be modulated by opposing mediators (e.g., IL-1β offset by IL-10 or RANTES), resulting in a balanced immune response and a stable clinical outcome. The lack of differences in psychiatric symptoms based on global inflammatory profiles, despite significant associations for selected cytokines (e.g., IL-1β, sIL-2Rα, IL-17, IL-10), highlights the need for composite immunological markers, such as the IL-10/TNF-α ratio, which better captures the pro-/anti-inflammatory balance and may offer more predictive value than individual cytokine levels. Similar strategies have been used in previous studies on treatment-resistant depression, PTSD, and chronic fatigue syndrome, where cytokine ratios or interaction patterns proved more sensitive predictors than absolute cytokine levels [106].

The findings suggest that cytokine dysregulation and inflammation may play a predisposing rather than determining role in the development of psychiatric symptoms. Symptom development likely results from a complex interplay of biological, psychological, and environmental factors—including individual resilience, perceived stress levels, and the presence or absence of additional burdens. This aligns with the “three-hit hypothesis”, a conceptual model in which the development of conditions such as depressive-anxiety disorders or schizophrenia is influenced by an interaction of (1) genetic vulnerability (first hit), (2) early environmental factors, such as childhood trauma (second hit), and (3) later-life stressors or environmental insults (third hit) [107,108]. Cytokine effects in relation to psychiatric disorders appear to be context-dependent and not necessarily reflected by the average inflammatory profile in serum. It is important to underscore that the classification of certain cytokines as “pro-inflammatory” does not automatically imply a detrimental effect on mental health or overall patient well-being. Under specific conditions, particularly at appropriate concentrations and temporal stages of infection, some cytokines may in fact exert neuroprotective or homeostatic functions, mitigating pathogen-induced damage. This has been observed for cytokines such as TNF-α, IL-12(p40), RANTES, MIP-1α, and IL-8. Thus, the severity of viral illness and immune activation do not directly predict psychiatric outcomes. This aligns with other studies on COVID-19, which also highlight the multifactorial origins of psychiatric symptoms after viral infections [44]. Furthermore, the variability in the predictive value of individual cytokines observed in our study, as well as the inconsistency with findings from other works, suggests that cytokines considered in isolation are unreliable biomarkers for psychiatric disorders. Inflammation and viral infections likely represent only partial components of the complex etiopathogenesis of mental illness, and no single causal pathway is expected to account for their development.

To advance the current understanding, future research should adopt integrative methods, e.g., proteomic, metabolomic, and genetic approaches, aligned with modern immunological insights. Emerging models like the neuroimmune-metabolic-oxidative (NIMETOX) pathway (Maes et al.) emphasize the interplay of neuroimmune, metabolic, and oxidative stress dysregulation in disorders such as MDD, bipolar disorder, and schizophrenia and should be considered in the development of updated pathophysiological models of psychiatric disorders [109]. Their framework moves beyond viewing depression as purely inflammatory, focusing instead on imbalances between Immune-Inflammatory Response System (IRS), Compensatory Immune Response System (CIRS), and Oxidative and Nitrosative Stress (O&NS). In line with our findings, they also advise against relying on isolated biomarkers, advocating for composite indicators instead [106,110].

## 4. Materials and Methods

### 4.1. Study Population

The study was conducted at the Department of Psychiatry, University Clinical Hospital in Bialystok, between February and December 2023. Patients hospitalized due to SARS-CoV-2, TBEV, or HCV infections were recruited from local infectious disease wards. Controls were healthy volunteers from hospital staff and the Healthy Senior University Program. Initially, 45 patients and 32 controls were enrolled; 8 patients were lost to follow-up, yielding a final sample of 37 patients (14 women, 23 men) and 32 controls (17 women, 15 men). The reasons for the lack of follow-up in eight patients were withdrawal of consent for further participation (6 patients) and inability to contact them (2 patients). The study cohort comprised 17 patients with COVID-19, 7 with HCV, and 13 with TBEV. All HCV patients were hospitalized for the first time; however, the duration of infection remained unknown. All participants were of Polish nationality and Caucasian ethnicity.

Inclusion criteria: age 18–90 years, current hospitalization due to one of the infections (patients), or no infection within the past year (controls), and mental capacity to complete psychiatric assessments.

Exclusion criteria: severe systemic illness, active cancer (cancer in remission was not an exclusion criterion for the study), schizophrenia, bipolar disorder, pregnancy/lactation, or lack of informed consent. Severe systemic illness was defined as a medical condition associated with significant organ failure, active malignancy, severe autoimmune disease, or other conditions requiring intensive or immunosuppressive treatment that could substantially affect immune function or psychiatric assessment.

### 4.2. Study Design

This was a 12-month prospective cohort study with two assessment points. At Stage 1 (during hospitalization), participants underwent psychiatric evaluation, mental state examination, and blood sampling. The Hospital Anxiety and Depression Scale (HADS) was administered. Control participants followed an identical protocol. ICD-10 diagnostic criteria were used throughout. At Stage 2 (after 12 months), follow-up interviews were conducted by phone, including HADS and an updated clinical interview. Psychiatric diagnoses were revised or confirmed by a senior psychiatrist.

### 4.3. Blood Collection and Sample Handling

During Stage 1, fasting venous blood (7.5 mL) was collected using S-Monovette^®^ Serum CAT tubes (SARSTEDT AG & Co. KG, Nümbrecht, Germany) Samples were left at room temperature for 45 min, centrifuged (5 °C, 2000× *g*, 10 min), and serum was transferred to 1.5 mL Eppendorf tubes (Eppendorf, Hamburg, Germany), then stored at −80 °C for up to 12 months.

### 4.4. Psychiatric Assessment

Participants completed a detailed interview covering demographics, comorbidities, medications, substance use, prior infections, psychiatric history, and vaccination status. In the study group, 12 participants (32.4%) had a prior psychiatric diagnosis—3 with anxiety disorders, 3 with mood disorders, and 4 with substance use disorders; 2 participants were unable to confirm the exact diagnosis. In the control group, 13 participants (40.6%) had a prior psychiatric diagnosis—6 with mood disorders and 5 with anxiety disorders; 1 participant was unable to confirm the exact diagnosis. Among the 25 individuals with a prior psychiatric diagnosis, only 2 (both with substance use disorder) had received treatment for it within the past 5 years. Current psychiatric symptoms (affective, anxiety, psychotic, obsessive-compulsive, cognitive, and sleep-related) were assessed based on ICD-10. Diagnoses were confirmed by an experienced psychiatrist (N.W.) in both study stages.

### 4.5. Scales

HADS was used at both stages to assess anxiety and depression (most common psychiatric disorders related to viral infections). The scale includes 14 items across two subscales (max score 21 each). Scores > 7 indicated clinical symptoms. At follow-up, HADS was administered via phone. The tool was selected for its brevity and diagnostic sensitivity [111,112].

### 4.6. Cytokine and Chemokine Measurement

After collection, all serum samples were thawed and analyzed simultaneously using the Bio-Plex Pro Human Cytokine Screening Panel, 48-Plex (#12007283) (Bio-Rad Laboratories, Hercules, CA, USA). This multiplex immunoassay quantifies cytokines, chemokines, and growth factors using fluorescently labeled magnetic beads. Assays were conducted per manufacturer protocol on the Bio-Plex 200 System (Bio-Rad Laboratories, Hercules, CA, USA). The full list of measured cytokines and chemokines is provided in the Supplementary Materials.

### 4.7. Statistical Analysis

Data analysis was performed using IBM SPSS Statistics version 30. Descriptive statistics and the Shapiro–Wilk test were used to evaluate distribution. Group comparisons were made with chi-square tests, Kruskal–Wallis tests, and Student’s *t*-tests, as appropriate. When the Kruskal–Wallis test indicated statistically significant differences, Dunn’s post hoc tests with Bonferroni correction were performed to examine pairwise comparisons. In analyses involving multiple cytokines, false discovery rate (FDR) correction according to the Benjamini–Hochberg procedure was additionally applied to control for multiple testing. Hierarchical logistic regression estimated event probabilities. Significance was set at α = 0.05; effect sizes were interpreted per Cohen’s guidelines [113].

Two approaches were applied to estimate the required sample size for this study: one for between-group comparisons and another for regression models.

For between-group comparisons, a priori power analysis conducted in G*Power version 3.1 indicated that, assuming a moderate effect size, a Type I error rate (α) of 0.05, and a statistical power of 0.95, the required sample size would be N = 280. For a conventional power level of 0.80, the required sample size would decrease to N = 180.

For regression analyses, a widely accepted rule of thumb was applied, recommending a minimum of 50 observations plus an additional 15 observations for each included predictor. Accordingly, a model with one predictor would require at least N = 50 participants, two predictors N = 65, and three predictors N = 80. Furthermore, due to the relatively large number of predictors compared to the sample size, regression analyses were performed using the backward elimination method to obtain the best-fitting model while excluding variables with low predictive power.

As the final step of the analysis, cluster analysis was performed. A two-step clustering approach based on the maximum likelihood method was first applied to automatically determine the optimal number of clusters, with solution quality assessed using the Silhouette measure. To verify the stability of the clustering solution, k-means cluster analysis was subsequently conducted using the number of clusters identified in the two-step procedure, and observations were assigned by minimizing within-cluster variance. All analyzed plasma parameters were included. Prior to clustering, data were standardized (z-scores) to ensure comparability across variables. The resulting clusters were interpreted as plasma cytokine profiles representing distinct biological patterns in the study population.

As the target sample size was not reached, the analyses were considered exploratory in nature and aimed primarily at generating hypotheses for future research.

## 5. Conclusions

In this study we did not find evidence supporting a direct impact of viral infection on the development of psychiatric disorders. Moreover, no dominant role of SARS-CoV-2 in psychiatric disorder development was observed, nor were there clear differences in symptom prevalence between infections. Despite distinct cytokine profiles and neurotropic properties, no virus-specific patterns of psychiatric diagnoses emerged. While some cytokines were linked to symptom risk, these associations were inconsistent with clinical presentation and insufficient as standalone biomarkers. Given the exploratory design and limited sample size, the findings underscore the multifactorial nature of post-viral psychiatric symptoms, shaped by immunological, psychological, and environmental factors. A linear “cytokine → symptom” model appears inadequate, and based on present preliminary data and current knowledge, it is not feasible to use a single time-point measurement of inflammatory cytokines in the blood to predict the emergence of psychiatric disorders. Future research, with larger, adequately powered cohorts, should adopt a systems-based approach, integrating composite markers and models like NIMETOX and IRS/CIRS/O&NS to better capture the complexity of psychiatric pathophysiology.

## 6. Limitations

This study has several limitations. Firstly, both the total sample size and subgroup sizes were small, reducing statistical power and restricting the ability to draw causal inferences and the generalizability of the results to broader populations until validated in larger, adequately powered cohorts. Therefore, the findings of the study should be interpreted as exploratory and considered a basis for generating of further hypotheses. Additionally, a statistically significant difference in age was observed between the TBEV and COVID-19 groups, which may have influenced the results. There were also differences in substance use and comorbidities across groups. The study did not account for detailed clinical indicators of infection severity, hospitalization course, and treatment-related factors. While all included infections were clinically mild to moderate, the lack of granular severity measures represents an important limitation. The only indicator of infection severity that can be objectively assessed by the reader is the level of inflammatory cytokines, which is available in a table in the external repository. Data on medication use, social support, socioeconomic status, and individual coping strategies were also not collected. Biomarkers were measured at a single time point, limiting insight into inflammatory dynamics. Using both HADS and subjective symptom ratings may have introduced inconsistencies. COVID-19 history was based on self-report without antibody testing, possibly missing asymptomatic cases. The high number of analyses increases the risk of type I error. Some limitations, especially group differences, reflect patterns seen in the general population, making the study more representative of real-world clinical samples. Even though the single-time-point cytokine measurement was intentional and aimed at testing its predictive value for post-viral psychiatric outcomes, it did not allow to capture the full dynamics of the immune response in infected patients and remains a key limitation of this study. Nonetheless, the present study illustrates the methodological challenges inherent in comparative viral research and highlights the value of pilot studies in identifying relevant variables and refining future study designs.

## Figures and Tables

**Table 1:** Results of Dunn’s post hoc test for anxiety and depression severity across diagnostic groups (Stage 2) assessed by the HADS. Each row tests the null hypothesis that the distributions of Group 1 and Group 2 are the same. Asymptotic significance values (two-tailed tests) are presented. The level of significance is 0.05. Significance values for multiple tests have been adjusted using the Bonferroni method.

Group 1–Group 2	SE	z	*p*	adj. *p*
TBEV—Control	6.536	1.738	0.082	0.494
TBEV—HCV	9.316	2.263	0.024	0.142
TBEV—COVID-19	7.321	2.909	0.004	0.022 *
Control—HCV	8.292	−1.173	0.241	1
Control—COVID-19	5.964	−1.667	0.095	0.573
HCV—COVID-19	8.924	0.024	0.98	1

Abbreviations: adj. *p*—adjusted *p*-value, COVID-19—coronavirus disease 2019, HCV—hepatitis C virus, *p*—*p*-value, SE—standard error, TBEV—Tick-borne encephalitis virus, z—standardized test statistics, *—statistical significance.

**Table 2:** Comparison of patients differentiated by diagnosis in terms of the levels of individual serum cytokines and chemokines, with False Discovery Rate correction (Benjamini–Hochberg) for multiple comparisons.

	Control Group (*n* = 32)			COVID-19 (*n* = 17)			HCV (*n* = 7)			TBEV (*n* = 13)						
Dependent Variable	Mean Rank	*M*	*SD*	Mean Rank	*M*	*SD*	Mean Rank	*M*	*SD*	Mean rank	*M*	*SD*	*H*(3)	*p*	Adj. *p*	η^2^
Eotaxin	35.03	124.79	55.43	32.88	118.48	60.50	35.29	123.21	42.00	37.54	134.93	73.85	0.40	0.940	0.975	<0.01
G-CSF	28.48	118.71	59.87	42.82	181.81	99.68	40.64	151.14	43.75	37.77	159.18	95.65	6.78	0.079	0.111	0.05
IFN-α2	33.03	1.40	0.48	32.00	1.31	0.00	42.00	6.90	13.61	40.00	2.79	3.29	9.83	**0.020**	**0.035**	0.11
IFN-γ	21.97	30.83	13.72	44.71	49.48	12.69	49.79	58.90	24.74	46.42	55.34	25.18	25.51	**<0.001**	**<0.001**	0.35
IL-1α	23.13	20.61	8.02	41.09	31.02	10.33	50.29	36.92	10.37	48.04	40.19	22.17	22.65	**<0.001**	**<0.001**	0.30
IL-1β	29.88	6.49	2.20	40.06	7.48	1.86	43.79	8.56	3.43	36.27	7.71	4.26	4.60	0.204	0.238	0.02
IL-1ra	22.70	102.52	130.79	45.82	415.32	434.37	43.50	335.95	285.46	46.54	357.61	232.60	22.75	**<0.001**	**<0.001**	0.30
IL-2	33.59	2.58	0.61	30.59	2.32	1.19	44.86	3.55	1.83	38.92	3.39	2.40	4.80	0.187	0.228	0.03
IL-2Rα	22.66	47.37	19.22	53.00	110.95	56.89	46.79	92.48	45.28	35.50	64.93	32.74	28.23	**<0.001**	**<0.001**	0.39
IL-4	23.88	2.05	0.71	40.26	2.64	0.63	50.21	3.15	0.72	47.31	3.36	1.70	20.14	**<0.001**	**<0.001**	0.26
IL-6	22.94	2.02	2.20	48.56	6.82	6.23	39.14	9.51	15.36	44.73	7.45	9.31	22.81	**<0.001**	**<0.001**	0.30
IL-7	28.77	32.16	11.94	34.06	36.79	16.89	46.79	44.93	13.75	45.23	46.46	23.16	9.14	**0.027**	**0.045**	0.09
IL-8	29.17	7.53	3.48	49.56	13.89	9.75	47.36	32.27	39.11	23.65	23.56	52.12	18.52	**<0.001**	**<0.001**	0.24
IL-9	37.31	649.11	83.60	31.41	629.68	65.62	38.21	657.20	35.03	32.27	594.34	180.86	1.39	0.708	0.762	<0.01
IL-10	21.63	5.00	3.95	43.21	15.31	15.09	46.64	13.84	6.61	50.92	19.10	12.64	27.97	**<0.001**	**<0.001**	0.38
IL-12(p70)	31.06	1.89	0.64	32.68	2.66	2.44	42.43	15.49	28.80	43.73	6.24	6.89	5.45	0.142	0.180	0.04
IL-12(p40)	23.98	12.29	13.10	38.12	18.72	13.82	45.86	24.35	27.03	52.19	40.71	50.11	25.02	**<0.001**	**<0.001**	0.34
IL-17	24.56	11.04	3.17	36.97	14.02	5.99	55.71	21.78	10.64	46.96	16.60	6.52	21.21	**<0.001**	**<0.001**	0.28
IP-10	23.78	265.69	156.21	49.50	1043.41	992.17	47.93	708.49	504.95	36.69	416.48	250.56	21.89	**<0.001**	**<0.001**	0.29
MCP-1	23.41	19.52	10.05	41.09	36.32	19.79	51.07	56.54	32.39	46.92	58.61	47.75	21.35	**<0.001**	**<0.001**	0.28
M-CSF	25.20	11.54	5.39	53.06	29.00	18.29	41.93	16.65	4.87	31.77	14.89	10.30	22.59	**<0.001**	**<0.001**	0.30
MIP-1α	28.63	1.37	0.97	42.97	2.36	1.56	43.57	2.31	1.27	35.65	2.04	1.84	7.22	0.065	0.096	0.06
MIP-1β	38.55	261.72	29.01	29.50	251.51	26.37	40.00	267.18	14.95	30.77	239.43	72.48	3.29	0.349	0.391	<0.01
RANTES	26.86	9.22	1.48	34.15	10.10	3.27	43.86	12.57	3.98	51.38	14.39	6.62	15.33	**0.002**	**0.003**	0.19
SCGF-β	32.00	114.82	31.90	39.91	129.83	52.14	48.36	135.14	18.88	28.77	101.16	37.97	6.09	0.107	0.143	0.05
TNF-α	25.52	89.67	16.64	39.71	123.27	93.57	50.86	140.00	59.06	43.65	110.76	41.64	14.95	**0.002**	**0.003**	0.18
TNF-β	39.73	462.88	59.03	29.56	440.43	51.79	44.64	478.80	32.19	25.27	401.17	122.29	7.71	0.052	0.082	0.06

Annotation. *n*—number of observations; *M*—mean; *SD*—standard deviation; *H*—test statistic value; *p*—statistical significance (statistically significant *p* values are highlighted in bold); adj. *p*—Benjamini–Hochberg adjusted *p*-values; η^2^—effect size indicator; G-CSF—granulocyte colony-stimulating factor; IFN-α2—interferon alpha 2; IFN-γ—interferon gamma; IL-1α—interleukin 1 alpha; IL-1β—interleukin 1 beta; IL-1ra—interleukin-1 receptor antagonist; IL-2—interleukin 2; IL-2Rα—interleukin-2 receptor alpha (soluble alpha subunit); IL-4—interleukin 4; IL-6—interleukin 6; IL-7—interleukin 7; IL-8—interleukin 8; IL-9—interleukin 9; IL-10—interleukin 10; IL-12(p70)—interleukin 12, p70 heterodimer; IL-12(p40)—interleukin 12, p40 subunit; IL-17—interleukin 17; IP-10—interferon gamma-induced protein 10; MCP-1—monocyte chemoattractant protein 1; M-CSF—macrophage colony-stimulating factor; MIP-1α—macrophage inflammatory protein 1 alpha; MIP-1β—macrophage inflammatory protein 1 beta; RANTES—regulated on activation, normal T cell expressed and secreted; SCGF-β—stem cell growth factor beta; TNF-α—tumor necrosis factor alpha; TNF-β—tumor necrosis factor beta.

**Table 3:** Cytokine Profiles by Diagnosis: The dominant cytokines identified in patient groups diagnosed with COVID-19, HCV, and TBEV are presented below.

Group	Dominant Cytokines
COVID-19	IL-10, IL-1ra, IL-2Rα, IL-6, IL-8, IP-10, M-CSF
HCV	IFN-γ, IL-10, IL-12(p40), IL-17, IL-1α, IP-10, TNF-α
TBEV	IL-12(p40), IL-17, IL-1ra, IL-1α, IL-4, IL-6, RANTES, TNF-α

Annotation: IFN-γ—interferon gamma; IL-1α—interleukin 1 alpha; IL-1ra—interleukin 1 receptor antagonist; IL-2Rα—interleukin 2 receptor alpha (soluble alpha subunit); IL-4—interleukin 4; IL-6—interleukin 6; IL-8—interleukin 8; IL-10—interleukin 10; IL-12(p40)—interleukin 12, p40 subunit; IL-17—interleukin 17; IP-10—interferon gamma-induced protein 10; M-CSF—macrophage colony-stimulating factor; RANTES—regulated upon activation, normal T cell expressed and secreted; TNF-α—tumor necrosis factor alpha.

**Table 4:** Summary of predictive findings—Stage 1 vs. Stage 2 (Based on cytokine levels measured in Stage 1). The table below presents a summary of cytokines that were significant predictors of psychiatric symptoms in Stage 1 and Stage 2 of the study. The change in probability of symptom occurrence is expressed per unit increase in the respective cytokine level. For example, for anxiety (HADS) in Stage 1, an increase in IL-12(p40) by one unit is associated with a 5% increase in the risk of experiencing anxiety symptoms.

Symptom	Stage 1	Stage 2
Anxiety (HADS)	IL-12(p40) (↑ 5%)	none
Depression (HADS)	IL-7 (↑ 12%), IL-9 (↑ 1%), IP-10 (↓ <1%), RANTES (↓ <1%)	IL-2 (↓ 83%), IL-12(p40) (↑ 12%), SCGF-β (↓ <1%)
Sleep disturbances	none	IL-10 (↓ 11%)
Cognitive impairments	Eotaxin (↑ 5%), IL-2Rα (↑ 5%), RANTES (↑ <1%), IL-17 (↓ 76%)	IL-1β (↑ 98%), IL-2Rα (↑ 5%), IL-8 (↑ 8%), MIP-1α (↓ 74%), RANTES (↑ <1%), TNF-α (↓ 10%)
Decreased energy	IL-10 (↑ 12%), RANTES (↓ <1%)	none
Depressed mood	IP-10 (↓ <1%), RANTES (↓ <1%)	IL-12(p40) (↑ 4%)
Anxiety	IL-12(p40) (↑ 3%), IL-17 (↓ 16%)	RANTES (↑ <1%)

Annotation: ↑—increase, ↓—decrease; IL-1β—interleukin 1 beta; IL-2—interleukin 2; IL-2Rα—interleukin 2 receptor alpha (soluble alpha subunit); IL-7—interleukin 7; IL-8—interleukin 8; IL-9—interleukin 9; IL-10—interleukin 10; IL-12(p40)—interleukin 12, p40 subunit; IL-17—interleukin 17; IP-10—interferon gamma-induced protein 10; MIP-1α—macrophage inflammatory protein 1 alpha; RANTES—regulated upon activation, normal T cell expressed and secreted; SCGF-β—stem cell growth factor beta; TNF-α—tumor necrosis factor alpha.

## Data Availability

Data available in a publicly accessible repository. The data presented in the study are openly available in FigShare at DOI: 10.6084/m9.figshare.30939122.

## Supplementary Materials

The following supporting information can be downloaded at: https://www.mdpi.com/article/10.3390/ijms27041670/s1.
