# Supplementary material for: Divergent Inflammatory Profiles but No Predictive Biomarkers of Psychiatric Sequelae After Viral Infection: A 12-Month Cohort Study
Source: Int J Mol Sci. 2026 Feb 9;27(4):1670. doi: 10.3390/ijms27041670 (PMC12940611; doi:10.3390/ijms27041670)
Supplement: Supplementary file 1 [file ijms-27-01670-s001.zip › ijms-4087943-supplementary.pdf]

Table S1. Comparison of patients differentiated by diagnosis in terms of sex, COVID-19 vaccination status, and history of psychiatric disorders

		Control group		Covid-19		HCV		TBEV				
		N	%	*N*	%	*N*	%	*N*	%	$\chi^2(3)$	*p*	Vc
Sex	Women	17	53,1%	6	35,3%	2	28,6%	6	46,2%	2,27	0,518	0,18
	Men	15	46,9%	11	64,7%	5	71,4%	7	53,8%			
Covid-19 vaccination	No	3	9,4%	2	11,8%	2	28,6%	2	15,4%	1,96	0,582	0,17
	Yes	29	90,6%	15	88,2%	5	71,4%	11	84,6%			
History of psychiatric disorders	No	19	59,4%	14	82,4%	4	57,1%	7	53,8%	3,50	0,321	0,23
	Yes	13	40,6%	3	17,6%	3	42,9%	6	46,2%			

Annotation. N – number of observations; χ^2 – chi-square test statistic; p – statistical significance; Vc – effect size indicator (Cramér's V).

Table S2. Comparison of patients differentiated by diagnosis in terms of age

Variable	Group	Mean rank	*M*	*SD*	*H*(3)	*p*	η^2
Age	Control group (*n* = 32)	37,06	55,84	17,30	15,04	**0,002**	0,19
	Covid-19 (*n* = 17)	46,06	64,88	13,49			
	HCV (*n* = 7)	29,79	52,43	10,50			
	TBEV (*n* = 13)	18,27	42,23	10,08			

Annotation. n – number of observations; M – mean; SD – standard deviation; H – test statistic value; p – statistical significance; η^2 – effect size indicator.

Table S3. Comparison of patients differentiated by diagnosis in terms of substance use

		Control group		Covid-19		HCV		TBEV				
Substance use		*N*	%	*N*	%	*N*	%	*N*	%	$\chi^2(3)$	*p*	Vc
Cigarettes	No	24	75,0%	14	82,4%	1	14,3%	8	61,5%	11,88	**0,008**	0,41
	Yes	8	25,0%	3	17,6%	6	85,7%	5	38,5%			
Alcohol	No	28	87,5%	15	88,2%	4	57,1%	11	84,6%	4,29	0,232	0,25
	Yes	4	12,5%	2	11,8%	3	42,9%	2	15,4%			
Drugs	No	30	93,8%	17	100,0%	5	71,4%	12	92,3%	6,12	0,106	0,29
	Yes	2	6,3%	0	0,0%	2	28,6%	1	7,7%			

Annotation. N – number of observations; χ^2 – chi-square test statistic; p – statistical significance; Vc – effect size indicator (Cramér's V).

Table S4. Comparison of patients differentiated by diagnosis in terms of comorbidities.

		Control Group		Covid-19		HCV		TBEV				
Comorbidities		*N*	%	*N*	%	*N*	%	*N*	%	$\chi^2(3)$	*p*	Vc
Diabetes	No	28	87,5%	13	76,5%	6	85,7%	13	100,0%	3,62	0,306	0,23
	Yes	4	12,5%	4	23,5%	1	14,3%	0	0,0%			

Hypertension	No	20	62,5%	8	47,1%	5	71,4%	11	84,6%	4,70	0,195	0,26
	Yes	12	37,5%	9	52,9%	2	28,6%	2	15,4%			
Heart diseases	No	30	93,8%	11	64,7%	7	100,0%	13	100,0%	12,83	**0,005**	0,43
	Yes	2	6,3%	6	35,3%	0	0,0%	0	0,0%			
Cancer in remission	No	31	96,9%	12	70,6%	7	100,0%	13	100,0%	12,35	**0,006**	0,42
	Yes	1	3,1%	5	29,4%	0	0,0%	0	0,0%			
Asthma/COPD	No	32	100,0%	15	88,2%	7	100,0%	12	92,3%	4,37	0,224	0,25
	Yes	0	0,0%	2	11,8%	0	0,0%	1	7,7%			
Hyperlipidemia	No	22	68,8%	16	94,1%	7	100,0%	13	100,0%	10,67	**0,014**	0,39
	Yes	10	31,3%	1	5,9%	0	0,0%	0	0,0%			

Annotation. N – number of observations; χ^2 – chi-square test statistic; p – statistical significance; Vc – effect size indicator (Cramér's V).

Table S5. Descriptive statistics of HADS scores in the study and control groups.

Dependent variable	*M*	*Me*	*SD*	*Sk.*	*Kurt.*	*Min.*	*Max.*	*W*	*p*
**Study group (*n* = 37)**									
Anxiety – stage 1	5,03	4,00	3,80	1,09	0,72	0,00	14,00	0,89	**0,001**
Depression - stage 1	4,76	4,00	5,04	1,29	0,73	0,00	18,00	0,82	**<0,001**
Anxiety - stage 2	4,86	4,00	3,66	1,05	1,33	0,00	16,00	0,92	**0,010**
Depression - stage 2	4,11	2,00	4,56	1,49	1,53	0,00	17,00	0,81	**<0,001**
Anxiety - change	-0,16	0,00	2,78	-0,26	1,03	-7,00	7,00	0,96	0,152
Depression - change	-0,65	0,00	4,42	-0,38	2,67	-14,00	12,00	0,94	**0,036**
**Control group (*n* = 32)**									
Anxiety - stage 1	6,13	6,00	4,10	0,62	-0,43	0,00	15,00	0,94	0,075
Depression - stage 1	5,03	3,00	4,62	0,83	-0,41	0,00	15,00	0,88	**0,001**
Anxiety - stage 2	4,78	5,00	2,38	0,26	-0,45	0,00	9,00	0,95	0,109
Depression - stage 2	3,00	2,00	2,72	1,04	0,42	0,00	10,00	0,89	**0,002**
Anxiety - change	-1,34	0,00	3,23	-1,54	2,64	-12,00	2,00	0,84	**<0,001**
Depression - change	-2,03	-1,00	3,41	-1,54	2,30	-13,00	2,00	0,82	**<0,001**

Annotation. M – mean; Me – median; SD – standard deviation; Sk. – skewness; Kurt. – kurtosis; Min. – minimum value; Max. – maximum value; W – Shapiro–Wilk test statistic; p – statistical significance for the Shapiro–Wilk test.

Table S5A. Descriptive statistics of HADS scores across viral subgroups (COVID-19, HCV, TBEV) and controls.

Dependent variable	*M*	*Me*	*SD*	*Sk.*	*Kurt.*	*Min.*	*Maks.*	*W*	*p*
**Covid-19 (*n* = 17)**									
Anxiety – stage 1	5,24	4,00	4,01	1,16	1,18	0,00	14,00	0,86	0,017
Depression - stage 1	6,06	4,00	5,18	0,73	-0,46	0,00	16,00	0,89	0,042
Anxiety - stage 2	5,65	5,00	4,11	0,99	1,48	0,00	16,00	0,91	0,117
Depression - stage 2	5,94	4,00	5,48	0,87	-0,38	0,00	17,00	0,89	0,041
Anxiety - change	0,41	0,00	2,32	-0,40	0,07	-4,00	4,00	0,94	0,285
Depression - change	-0,12	0,00	4,44	0,94	2,61	-8,00	12,00	0,92	0,160
**HCV (*n* = 7)**									
Anxiety – stage 1	6,43	7,00	4,58	0,33	0,15	0,00	14,00	0,98	0,964
Depression - stage 1	4,71	3,00	6,05	2,30	5,72	0,00	18,00	0,69	0,002
Anxiety - stage 2	5,00	4,00	3,56	1,49	2,15	2,00	12,00	0,85	0,118
Depression - stage 2	4,71	4,00	3,82	1,29	1,51	1,00	12,00	0,88	0,246
Anxiety - change	-1,43	-1,00	2,99	-1,04	1,28	-7,00	2,00	0,94	0,630
Depression - change	0,00	-1,00	4,08	-0,12	-1,44	-6,00	5,00	0,94	0,613
**TBEV (*n* = 13)**									

Anxiety – stage 1	4,00	3,00	3,00	1,73	3,55	1,00	12,00	0,83	0,016
Depression - stage 1	3,08	2,00	4,09	1,99	3,87	0,00	14,00	0,74	0,001
Anxiety - stage 2	3,77	4,00	3,03	0,75	0,02	0,00	10,00	0,92	0,260
Depression - stage 2	1,38	1,00	1,39	0,50	-0,97	0,00	4,00	0,87	0,051
Anxiety - change	-0,23	0,00	3,19	0,27	1,91	-6,00	7,00	0,91	0,214
Depression - change	-1,69	0,00	4,71	-1,98	3,42	-14,00	2,00	0,71	**<0,001**
**Control group (*n* = 32)**									
Anxiety – stage 1	6,13	6,00	4,10	0,62	-0,43	0,00	15,00	0,94	0,075
Depression - stage 1	5,03	3,00	4,62	0,83	-0,41	0,00	15,00	0,88	**0,001**
Anxiety - stage 2	4,78	5,00	2,38	0,26	-0,45	0,00	9,00	0,95	0,109
Depression - stage 2	3,00	2,00	2,72	1,04	0,42	0,00	10,00	0,89	**0,002**
Anxiety - change	-1,34	0,00	3,23	-1,54	2,64	-12,00	2,00	0,84	**<0,001**
Depression - change	-2,03	-1,00	3,41	-1,54	2,30	-13,00	2,00	0,82	**<0,001**

Annotation. M – mean; Me – median; SD – standard deviation; Sk. – skewness; Kurt. – kurtosis; Min. – minimum value; Max. – maximum value; W – Shapiro–Wilk test statistic; p – statistical significance for the Shapiro–Wilk test.

Table S6. Descriptive statistics of plasma parameters in the study and control groups

Dependent variable	*M*	*Me*	*SD*	*Sk.*	*Kurt.*	*Min.*	*Max.*	*W*	*p*
**Study group (*n* = 37)**									
Eotaxin	125,15	112,85	61,60	0,94	1,47	1,73	305,59	0,92	**0,009**
G-CSF	168,06	139,69	89,21	1,26	1,48	42,74	414,04	0,89	**0,001**
IFN-α2	2,89	1,31	6,23	5,21	28,86	1,31	37,68	0,28	**<0,001**
IFN-γ	53,32	48,90	19,98	0,67	1,03	8,06	104,23	0,95	0,105
IL-1α	35,36	32,70	15,72	1,29	3,61	3,95	92,19	0,90	**0,003**
IL-1β	7,77	7,41	3,12	0,90	2,13	0,52	17,02	0,93	**0,017**
IL-1ra	369,22	254,80	300,70	1,34	1,95	9,57	1353,56	0,89	**0,001**
IL-2	2,88	2,77	1,64	1,32	3,77	0,70	8,77	0,82	**<0,001**
IL-2Rα	91,29	71,64	50,71	1,50	3,33	5,19	273,50	0,87	**<0,001**
IL-4	2,99	2,72	1,16	1,25	5,16	0,18	7,34	0,87	**<0,001**
IL-6	6,87	4,37	7,03	1,96	3,22	0,50	28,00	0,73	**<0,001**
IL-7	41,73	38,83	18,94	0,64	0,70	3,22	89,25	0,95	0,077
IL-8	16,17	8,91	17,59	1,99	3,06	1,27	72,00	0,68	**<0,001**
IL-9	630,58	636,12	81,55	-1,13	2,18	361,56	751,57	0,92	**0,012**
IL-10	16,36	12,45	12,89	1,76	2,95	4,49	58,82	0,79	**<0,001**
IL-12 (p70)	4,45	2,11	4,80	1,54	1,97	0,39	19,77	0,79	**<0,001**
IL-12 (p40)	24,81	11,56	21,96	1,73	2,65	10,00	95,46	0,71	**<0,001**
IL-17	16,39	13,73	7,57	1,65	2,61	4,27	37,55	0,80	**<0,001**
IP-10	730,04	517,41	671,10	1,95	3,46	10,74	2673,92	0,76	**<0,001**
MCP-1	47,98	35,79	35,05	1,71	3,48	6,39	165,95	0,84	**<0,001**
M-CSF	21,71	17,74	15,33	1,97	4,73	0,76	75,35	0,82	**<0,001**
MIP-1α	2,24	1,91	1,58	1,26	1,88	0,05	6,89	0,90	**0,002**
MIP-1β	254,56	256,80	28,04	-1,04	1,28	173,75	300,55	0,92	**0,015**
RANTES (thous.)	12,08	11,63	5,08	0,56	1,67	0,20	26,91	0,95	0,095
SCGF-β (thous.)	120,76	123,59	44,34	0,21	3,18	0,71	258,87	0,92	**0,013**
TNF-α	116,09	106,66	45,22	1,90	4,50	30,39	263,95	0,76	**<0,001**
TNF-β	440,11	452,88	58,22	-1,38	2,11	257,73	520,63	0,89	**0,001**
**Control group (*n* = 32)**									
Eotaxin	124,79	117,02	55,43	1,09	1,79	45,37	299,08	0,93	**0,049**
G-CSF	118,71	112,80	59,87	1,13	2,17	42,74	313,95	0,92	**0,015**
IFN-α2	1,40	1,31	0,48	5,66	32,00	1,31	4,05	0,17	**<0,001**
IFN-γ	30,83	27,92	13,72	1,23	1,30	13,00	68,92	0,89	**0,003**
IL-1α	20,61	18,16	8,02	0,52	-0,52	8,63	37,59	0,93	**0,048**

Dependent variable	*M*	*Me*	*SD*	*Sk.*	*Kurt.*	*Min.*	*Max.*	*W*	*p*
IL-1β	6,49	6,16	2,20	0,82	1,31	2,61	12,58	0,94	0,080
IL-1ra	102,52	28,74	130,79	2,00	4,46	9,57	570,06	0,73	**<0,001**
IL-2	2,58	2,77	0,61	-2,93	7,00	0,70	2,77	0,33	**<0,001**
IL-2Rα	47,37	42,90	19,22	1,06	0,99	21,70	101,79	0,91	**0,012**
IL-4	2,05	1,86	0,71	0,89	0,27	1,08	3,65	0,90	**0,008**
IL-6	2,02	1,03	2,20	2,10	5,31	0,44	10,11	0,74	**<0,001**
IL-7	32,16	32,02	11,94	0,71	0,02	17,07	57,81	0,90	**0,006**
IL-8	7,53	6,86	3,48	3,46	15,71	3,92	23,82	0,66	**<0,001**
IL-9	649,11	668,31	83,60	-0,75	0,61	432,27	791,95	0,96	0,212
IL-10	5,00	4,49	3,95	0,94	0,06	0,13	15,03	0,86	**<0,001**
IL-12 (p70)	1,89	2,11	0,64	-1,70	2,08	0,39	3,00	0,56	**<0,001**
IL-12 (p40)	12,29	10,00	13,10	5,47	30,64	0,46	83,35	0,24	**<0,001**
IL-17	11,04	10,57	3,17	0,64	-0,05	6,63	18,48	0,92	**0,024**
IP-10	265,69	212,57	156,21	1,20	0,64	54,60	677,65	0,86	**<0,001**
MCP-1	19,52	17,03	10,05	1,37	1,83	7,01	50,70	0,88	**0,001**
M-CSF	11,54	9,90	5,39	1,00	0,47	3,87	24,91	0,91	**0,011**
MIP-1α	1,37	1,34	0,97	0,61	0,11	0,05	3,88	0,95	0,140
MIP-1β	261,72	269,10	29,01	-0,89	0,06	192,81	306,67	0,92	**0,018**
RANTES (thous.)	9,22	9,50	1,48	-1,56	4,39	3,87	11,29	0,88	**0,002**
SCGF-β (thous.)	114,82	108,68	31,90	0,96	0,80	58,91	198,95	0,93	**0,038**
TNF-α	89,67	84,12	16,64	0,67	-0,14	59,12	130,32	0,94	0,055
TNF-β	462,88	475,84	59,03	-0,81	0,33	308,59	552,57	0,95	0,111

Annotation. M – mean; Me – median; SD – standard deviation; Sk. – skewness; Kurt. – kurtosis; Min. – minimum value; Max. – maximum value; W – Shapiro–Wilk test statistic; p – statistical significance for the Shapiro–Wilk test.

Table S6A. Descriptive statistics of plasma parameters across viral subgroups (COVID-19, HCV, TBEV) and controls.

Dependent variable	*M*	*Me*	*SD*	*Sk.*	*Kurt.*	*Min.*	*Maks.*	*W*	*p*
**Covid-19 (*n* = 17)**									
Eotaxin	118,48	106,93	60,50	1,82	5,60	18,58	305,59	0,83	**0,005**
G-CSF	181,81	146,34	99,68	0,91	0,21	50,03	407,83	0,93	0,190
IFN-α2	1,31	1,31	0,00			1,31	1,31		
IFN-γ	49,48	48,38	12,69	0,38	-0,68	31,26	74,23	0,96	0,667
IL-1α	31,02	27,83	10,33	0,28	-0,75	13,37	47,42	0,94	0,270
IL-1β	7,48	7,41	1,86	0,09	-0,95	4,57	10,78	0,97	0,765
IL-1ra	415,32	254,80	434,37	2,14	5,08	28,74	1753,56	0,75	**<0,001**
IL-2	2,32	2,77	1,19	-0,14	-0,23	0,70	4,83	0,78	**0,001**
IL-2Rα	110,95	95,11	56,89	1,55	3,02	46,87	273,50	0,87	**0,018**
IL-4	2,64	2,58	0,63	-0,04	3,17	1,08	4,16	0,91	0,092
IL-6	6,82	4,82	6,23	2,22	4,37	1,53	24,35	0,68	**<0,001**
IL-7	36,79	32,02	16,89	0,88	0,65	8,29	75,36	0,90	0,077
IL-8	13,89	10,28	9,75	2,74	8,51	6,17	46,83	0,67	**<0,001**
IL-9	629,68	636,12	65,62	-0,59	0,21	487,34	736,78	0,97	0,735
IL-10	15,31	12,45	15,09	2,20	4,53	4,49	58,82	0,68	**<0,001**
IL-12 (p70)	2,66	2,11	2,44	1,45	1,11	0,39	8,26	0,75	**<0,001**
IL-12 (p40)	18,72	11,56	13,82	1,45	0,80	10,00	48,94	0,66	**<0,001**
IL-17	14,02	13,73	5,99	1,84	5,93	4,27	32,77	0,83	**0,005**
IP-10	1043,41	643,52	992,17	1,50	1,08	82,45	3273,92	0,77	**<0,001**
MCP-1	36,32	30,73	19,79	1,05	0,63	15,72	85,05	0,88	**0,031**
M-CSF	29,00	22,67	18,29	1,60	2,18	10,12	75,35	0,82	**0,004**
MIP-1α	2,36	2,17	1,56	1,24	2,49	0,15	6,67	0,92	0,161
MIP-1β	251,51	256,12	26,37	-1,00	0,99	193,34	285,87	0,89	**0,042**
RANTES (thous.)	10,10	10,01	3,27	-0,26	0,43	3,78	15,81	0,94	0,298

Dependent variable	*M*	*Me*	*SD*	*Sk.*	*Kurt.*	*Min.*	*Maks.*	*W*	*p*
SCGF-β (thous.)	129,83	134,11	52,13	0,65	2,08	22,74	258,87	0,93	0,184
TNF-α	123,27	104,29	93,57	4,03	16,46	78,18	483,95	0,37	**<0,001**
TNF-β	440,43	450,38	51,79	-1,59	3,22	297,03	518,12	0,84	**0,008**
**HCV (*n* = 7)**									
Eotaxin	123,21	119,26	42,00	0,74	-0,19	78,13	195,54	0,92	0,491
G-CSF	151,14	152,98	43,75	-0,16	-2,15	99,18	198,88	0,87	0,173
IFN-α2	6,90	1,31	13,61	2,62	6,87	1,31	37,68	0,50	**<0,001**
IFN-γ	58,90	57,30	24,74	0,68	1,12	25,11	103,15	0,96	0,780
IL-1α	36,92	32,70	10,37	0,56	-1,74	27,83	52,35	0,84	0,100
IL-1β	8,56	8,34	3,43	1,62	3,24	5,21	15,55	0,85	0,118
IL-1ra	335,95	197,61	285,46	0,97	-0,63	87,72	810,41	0,85	0,127
IL-2	3,55	2,77	1,83	-0,09	-0,42	0,70	6,17	0,93	0,554
IL-2Rα	92,48	67,16	45,28	0,84	-1,16	51,39	163,89	0,85	0,113
IL-4	3,15	2,86	0,72	0,81	0,20	2,30	4,41	0,94	0,676
IL-6	9,51	3,44	15,36	2,28	5,29	1,03	43,00	0,64	**<0,001**
IL-7	44,93	45,37	13,75	0,61	1,65	24,82	69,63	0,94	0,678
IL-8	32,27	9,82	39,11	1,71	2,57	5,27	110,86	0,76	**0,015**
IL-9	657,20	652,21	35,03	0,74	-0,39	622,72	716,62	0,92	0,439
IL-10	13,84	12,45	6,61	0,36	-0,60	4,49	22,59	0,87	0,200
IL-12 (p70)	15,49	2,16	28,80	2,48	6,30	0,39	79,77	0,60	**<0,001**
IL-12 (p40)	24,35	11,56	27,03	2,30	5,33	10,00	83,35	0,61	**<0,001**
IL-17	21,78	16,89	10,64	1,13	-0,92	13,73	37,55	0,73	**0,007**
IP-10	708,49	555,28	504,95	1,58	2,47	214,82	1712,59	0,84	0,102
MCP-1	56,54	64,02	32,39	0,01	-1,53	17,03	102,18	0,94	0,614
M-CSF	16,65	16,39	4,87	1,35	2,53	11,47	26,26	0,88	0,248
MIP-1α	2,31	1,91	1,27	0,45	-1,62	0,89	4,15	0,92	0,449
MIP-1β	267,18	265,67	14,95	0,32	-0,32	247,15	291,12	0,99	0,982
RANTES (thous.)	12,57	10,41	3,98	0,52	-2,16	9,05	17,62	0,79	**0,032**
SCGF-β (thous.)	135,14	134,58	18,88	0,19	-0,42	112,19	165,11	0,94	0,615
TNF-α	140,00	111,40	59,06	1,56	1,33	97,18	252,70	0,73	**0,007**
TNF-β	478,80	492,14	32,19	-0,15	-1,96	439,54	520,63	0,90	0,329
**TBEV (*n* = 13)**									
Eotaxin	134,93	118,59	73,85	0,22	-0,45	1,73	251,32	0,94	0,451
G-CSF	159,18	119,56	95,65	1,66	3,51	42,74	414,04	0,84	**0,021**
IFN-α2	2,79	1,31	3,29	2,47	6,04	1,31	12,30	0,54	**<0,001**
IFN-γ	55,34	48,90	25,18	0,30	0,35	8,06	104,23	0,95	0,590
IL-1α	40,19	32,70	22,17	0,88	1,47	3,95	92,19	0,91	0,184
IL-1β	7,71	7,41	4,26	0,70	0,89	0,52	17,02	0,94	0,510
IL-1ra	357,61	305,90	232,60	0,15	-0,85	9,57	719,30	0,95	0,590
IL-2	3,39	2,77	2,40	2,69	8,56	0,70	10,77	0,66	**<0,001**
IL-2Rα	64,93	59,29	32,74	0,84	2,51	5,19	143,96	0,91	0,203
IL-4	3,36	3,13	1,70	0,63	2,05	0,18	7,34	0,94	0,421
IL-6	7,45	3,91	9,31	2,83	9,00	0,50	36,35	0,64	**<0,001**
IL-7	46,46	45,37	23,16	0,37	0,75	3,22	89,25	0,94	0,405
IL-8	23,56	5,27	52,12	3,29	11,15	1,27	192,00	0,45	**<0,001**
IL-9	594,34	636,12	180,86	-2,34	6,71	61,56	751,57	0,74	**0,001**
IL-10	19,10	15,03	12,64	0,92	-0,27	4,49	42,15	0,87	0,054
IL-12 (p70)	6,24	4,65	6,89	2,02	4,96	0,39	25,60	0,78	**0,003**
IL-12 (p40)	40,71	30,86	50,11	2,79	8,73	10,00	195,46	0,62	**<0,001**
IL-17	16,60	14,52	6,52	1,74	4,38	7,41	34,36	0,82	**0,012**
IP-10	416,48	423,43	250,56	0,41	-0,39	10,74	894,34	0,96	0,789
MCP-1	58,61	51,97	47,75	1,43	1,51	6,39	165,95	0,83	**0,017**

Dependent variable	*M*	*Me*	*SD*	*Sk.*	*Kurt.*	*Min.*	*Maks.*	*W*	*p*
M-CSF	14,89	13,48	10,30	1,16	1,38	0,76	38,84	0,91	0,208
MIP-1α	2,04	1,40	1,84	1,70	3,27	0,05	6,89	0,84	**0,022**
MIP-1β	239,43	252,99	72,48	-2,85	9,16	13,75	300,55	0,65	**<0,001**
RANTES (thous.)	14,39	14,17	6,62	-0,10	1,33	0,20	26,91	0,94	0,494
SCGF-β (thous.)	101,16	109,63	37,97	-1,57	3,39	0,71	147,71	0,88	0,062
TNF-α	110,76	116,13	41,64	0,37	1,99	30,39	205,74	0,94	0,495
TNF-β	401,17	437,88	122,29	-2,67	8,21	27,73	504,70	0,68	**<0,001**
**Control group (*n* = 32)**									
Eotaxin	124,79	117,02	55,43	1,09	1,79	45,37	299,08	0,93	**0,049**
G-CSF	118,71	112,80	59,87	1,13	2,17	42,74	313,95	0,92	**0,015**
IFN-α2	1,40	1,31	0,48	5,66	32,00	1,31	4,05	0,17	**<0,001**
IFN-γ	30,83	27,92	13,72	1,23	1,30	13,00	68,92	0,89	**0,003**
IL-1α	20,61	18,16	8,02	0,52	-0,52	8,63	37,59	0,93	**0,048**
IL-1β	6,49	6,16	2,20	0,82	1,31	2,61	12,58	0,94	0,080
IL-1ra	102,52	28,74	130,79	2,00	4,46	9,57	570,06	0,73	**<0,001**
IL-2	2,58	2,77	0,61	-2,93	7,00	0,70	2,77	0,33	**<0,001**
IL-2Rα	47,37	42,90	19,22	1,06	0,99	21,70	101,79	0,91	**0,012**
IL-4	2,05	1,86	0,71	0,89	0,27	1,08	3,65	0,90	**0,008**
IL-6	2,02	1,03	2,20	2,10	5,31	0,44	10,11	0,74	**<0,001**
IL-7	32,16	32,02	11,94	0,71	0,02	17,07	57,81	0,90	**0,006**
IL-8	7,53	6,86	3,48	3,46	15,71	3,92	23,82	0,66	**<0,001**
IL-9	649,11	668,31	83,60	-0,75	0,61	432,27	791,95	0,96	0,212
IL-10	5,00	4,49	3,95	0,94	0,06	0,13	15,03	0,86	**<0,001**
IL-12 (p70)	1,89	2,11	0,64	-1,70	2,08	0,39	3,00	0,56	**<0,001**
IL-12 (p40)	12,29	10,00	13,10	5,47	30,64	0,46	83,35	0,24	**<0,001**
IL-17	11,04	10,57	3,17	0,64	-0,05	6,63	18,48	0,92	**0,024**
IP-10	265,69	212,57	156,21	1,20	0,64	54,60	677,65	0,86	**<0,001**
MCP-1	19,52	17,03	10,05	1,37	1,83	7,01	50,70	0,88	**0,001**
M-CSF	11,54	9,90	5,39	1,00	0,47	3,87	24,91	0,91	**0,011**
MIP-1α	1,37	1,34	0,97	0,61	0,11	0,05	3,88	0,95	0,140
MIP-1β	261,72	269,10	29,01	-0,89	0,06	192,81	306,67	0,92	**0,018**
RANTES (thous.)	9,22	9,50	1,48	-1,56	4,39	3,87	11,29	0,88	**0,002**
SCGF-β (thous.)	114,82	108,68	31,90	0,96	0,80	58,91	198,95	0,93	**0,038**
TNF-α	89,67	84,12	16,64	0,67	-0,14	59,12	130,32	0,94	0,055
TNF-β	462,88	475,84	59,03	-0,81	0,33	308,59	552,57	0,95	0,111

Annotation. M – mean; Me – median; SD – standard deviation; Sk. – skewness; Kurt. – kurtosis; Min. – minimum value; Max. – maximum value; W – Shapiro–Wilk test statistic; p – statistical significance for the Shapiro–Wilk test.

Table S7. Comparison of patients differentiated by diagnosis in terms of the presence of specific psychiatric symptoms at Stage 1 of the study

Psychiatric symptoms at Stage 1		Control group		Covid-19		HCV		TBEV		$\chi^2(3)$	*p*	Vc
		N	%	*N*	%	*N*	%	*N*	%			
Anxiety- HADS	No	23	71,9%	14	82,4%	4	57,1%	12	92,3%	4,00	0,261	0,24
	Yes	9	28,1%	3	17,6%	3	42,9%	1	7,7%			
Depression - HADS	No	22	68,8%	12	70,6%	6	85,7%	11	84,6%	1,82	0,611	0,16
	Yes	10	31,3%	5	29,4%	1	14,3%	2	15,4%			
Sleep disorders	No	23	71,9%	14	82,4%	5	71,4%	12	92,3%	2,63	0,452	0,20
	Yes	9	28,1%	3	17,6%	2	28,6%	1	7,7%			

Cognitive impairment	No	28	87,5%	13	76,5%	7	100,0%	12	92,3%	3,04	0,386	0,21
	Yes	4	12,5%	4	23,5%	0	0,0%	1	7,7%			
Loss of energy	No	26	81,3%	13	76,5%	6	85,7%	12	92,3%	1,39	0,709	0,14
	Yes	6	18,8%	4	23,5%	1	14,3%	1	7,7%			
Depressed mood	No	20	62,5%	12	70,6%	6	85,7%	11	84,6%	3,03	0,387	0,21
	Yes	12	37,5%	5	29,4%	1	14,3%	2	15,4%			
Anxiety	No	21	65,6%	13	76,5%	5	71,4%	10	76,9%	0,92	0,821	0,12
	Yes	11	34,4%	4	23,5%	2	28,6%	3	23,1%			
Obsessions/compulsions	No	31	96,9%	16	94,1%	7	100,0%	13	100,0%	1,14	0,767	0,13
	Yes	1	3,1%	1	5,9%	0	0,0%	0	0,0%			

Annotation. N – number of observations; χ² – chi-square test statistic; p – statistical significance; Vc – effect size indicator (Cramér's V).

Table S8. Comparison of patients differentiated by diagnosis in terms of the presence of specific psychiatric symptoms at Stage 2 of the study

Psychiatric symptoms at Stage 2		Control group		Covid-19		HCV		TBEV		$\chi^2(3)$	*p*	Vc
		N	%	*N*	%	*N*	%	*N*	%			
Anxiety- HADS	No	27	84,4%	14	82,4%	6	85,7%	11	84,6%	0,06	0,996	0,03
	Yes	5	15,6%	3	17,6%	1	14,3%	2	15,4%			
Depression - HADS	No	29	90,6%	12	70,6%	6	85,7%	13	100,0%	6,36	0,096	0,30
	Yes	3	9,4%	5	29,4%	1	14,3%	0	0,0%			
Sleep disorders	No	23	71,9%	12	70,6%	4	57,1%	13	100,0%	5,92	0,116	0,29
	Yes	9	28,1%	5	29,4%	3	42,9%	0	0,0%			
Cognitive impairment	No	28	87,5%	12	70,6%	5	71,4%	7	53,8%	6,05	0,109	0,30
	Yes	4	12,5%	5	29,4%	2	28,6%	6	46,2%			
Loss of energy	No	26	81,3%	15	88,2%	6	85,7%	12	92,3%	1,06	0,788	0,12
	Yes	6	18,8%	2	11,8%	1	14,3%	1	7,7%			
Depressed mood	No	26	81,3%	14	82,4%	5	71,4%	11	84,6%	0,55	0,908	0,09
	Yes	6	18,8%	3	17,6%	2	28,6%	2	15,4%			
Anxiety	No	23	71,9%	14	82,4%	4	57,1%	9	69,2%	1,73	0,630	0,16
	Yes	9	28,1%	3	17,6%	3	42,9%	4	30,8%			
Obsessions/compulsions	No	31	96,9%	17	100,0%	7	100,0%	13	100,0%	1,17	0,759	0,13
	Yes	1	3,1%	0	0,0%	0	0,0%	0	0,0%			

Annotation. N – number of observations; χ² – chi-square test statistic; p – statistical significance; Vc – effect size indicator (Cramér's V).

Table S9. Comparison of patients differentiated by diagnosis in terms of changes in the presence of specific psychiatric symptoms over the course of the study

Change over the course of the study		Control group		Covid-19		HCV		TBEV		$\chi^2(6)$	*p*	Vc
		N	%	*N*	%	*N*	%	*N*	%			
	No change	24	75,0%	17	100,0%	5	71,4%	10	76,9%	9,06	0,170	0,26

Anxiety-HADS	Symptom remission	6	18,8%	0	0,0%	2	28,6%	1	7,7%			
	Symptom onset	2	6,3%	0	0,0%	0	0,0%	2	15,4%			
Depression - HADS	No change	25	78,1%	13	76,5%	7	100,0%	11	84,6%	8,54	0,201	0,25
	Symptom remission	7	21,9%	2	11,8%	0	0,0%	2	15,4%			
	Symptom onset	0	0,0%	2	11,8%	0	0,0%	0	0,0%			
Sleep disorders	No change	28	87,5%	13	76,5%	6	85,7%	12	92,3%	3,91	0,688	0,17
	Symptom remission	2	6,3%	1	5,9%	0	0,0%	1	7,7%			
	Symptom onset	2	6,3%	3	17,6%	1	14,3%	0	0,0%			
Cognitive impairment	No change	28	87,5%	14	82,4%	5	71,4%	8	61,5%	8,99	0,174	0,26
	Symptom remission	2	6,3%	1	5,9%	0	0,0%	0	0,0%			
	Symptom onset	2	6,3%	2	11,8%	2	28,6%	5	38,5%			
Loss of energy	No change	30	93,8%	13	76,5%	7	100,0%	11	84,6%	5,14	0,525	0,19
	Symptom remission	1	3,1%	3	17,6%	0	0,0%	1	7,7%			
	Symptom onset	1	3,1%	1	5,9%	0	0,0%	1	7,7%			
Depressed mood	No change	26	81,3%	11	64,7%	6	85,7%	11	84,6%	6,85	0,335	0,22
	Symptom remission	6	18,8%	4	23,5%	0	0,0%	1	7,7%			
	Symptom onset	0	0,0%	2	11,8%	1	14,3%	1	7,7%			
Anxiety	No change	28	87,5%	12	70,6%	6	85,7%	10	76,9%	4,44	0,618	0,18
	Symptom remission	3	9,4%	3	17,6%	0	0,0%	1	7,7%			
	Symptom onset	1	3,1%	2	11,8%	1	14,3%	2	15,4%			
Obsessions/compulsions	No change	32	100,0%	16	94,1%	7	100,0%	13	100,0%	3,10	0,376	0,21
	Symptom remission	0	0,0%	1	5,9%	0	0,0%	0	0,0%			
	Symptom onset	0	0,0%	0	0,0%	0	0,0%	0	0,0%			

Annotation. N – number of observations; χ^2 – chi-square test statistic; p – statistical significance; Vc – effect size indicator (Cramér's V).

Table S10. Comparison of patients differentiated by diagnosis in terms of anxiety and depression severity at Stage 1 of the study

Stage 1		Mean rank	*M*	*SD*	*H*(3)	*p*	η^2
Anxiety	Control group (*n* = 32)	38,00	6,13	4,10	3,66	0,300	0,01
	Covid-19 (*n* = 17)	33,91	5,24	4,01			
	HCV (*n* = 7)	40,00	6,43	4,58			
	TBEV (*n* = 13)	26,35	4,00	3,00			
Depression	Control group (*n* = 32)	35,77	5,03	4,62	4,36	0,225	0,02
	Covid-19 (*n* = 17)	40,97	6,06	5,18			
	HCV (*n* = 7)	34,07	4,71	6,05			
	TBEV (*n* = 13)	25,81	3,08	4,09			

Annotation. n – number of observations; M – mean; SD – standard deviation; H – test statistic value; p – statistical significance; η^2 – effect size indicator.

Table S11. Comparison of patients differentiated by diagnosis in terms of anxiety and depression severity at Stage 2 of the study

Stage 2		Mean rank	*M*	*SD*	*H*(3)	*p*	η²
Anxiety	Control group (*n* = 32)	35,92	4,78	2,38	2,68	0,443	<0,01
	Covid-19 (*n* = 17)	39,35	5,65	4,11			
	HCV (*n* = 7)	33,93	5,00	3,56			
	TBEV (*n* = 13)	27,62	3,77	3,03			
Depression	Control group (*n* = 32)	33,70	3,00	2,72	9,89	**0,020**	0,11
	Covid-19 (*n* = 17)	43,65	5,94	5,48			
	HCV (*n* = 7)	43,43	4,71	3,82			
	TBEV (*n* = 13)	22,35	1,38	1,39			

Annotation. n – number of observations; M – mean; SD – standard deviation; H – test statistic value; p – statistical significance; η² – effect size indicator.

Table S12. Comparison of patients differentiated by diagnosis in terms of changes in anxiety and depression severity between Stage 1 and Stage 2 of the study

Change over the course of the study		Mean rank	*M*	*SD*	*H*(3)	*p*	η²
Anxiety	Control group (*n* = 32)	31,97	-1,34	3,23	3,55	0,315	<0,01
	Covid-19 (*n* = 17)	42,21	0,41	2,32			
	HCV (*n* = 7)	29,64	-1,43	2,99			
	TBEV (*n* = 13)	35,92	-0,23	3,19			
Depression	Control group (*n* = 32)	30,88	-2,03	3,41	2,64	0,450	<0,01
	Covid-19 (*n* = 17)	39,03	-0,12	4,44			
	HCV (*n* = 7)	39,50	0,00	4,08			
	TBEV (*n* = 13)	37,46	-1,69	4,71			

Annotation. n – number of observations; M – mean; SD – standard deviation; H – test statistic value; p – statistical significance; η² – effect size indicator.

Table S13. Percentage and total number of Psychiatric Diagnoses by Group – Stage 1

Diagnosis	COVID-19 (n=17)	Control Group (n=32)	HCV (n=7)	TBEV (n=13)
f06.7	5.9 (1)	0.0	0.0	0.0
f10.2	0.0	3.1 (1)	0.0	0.0
f19.2	0.0	0.0	28.6 (2)	7.7 (1)
f32	5.9 (1)	6.2 (2)	14.3 (1)	23.1 (3)
f33	0.0	6.2 (2)	0.0	0.0
f41.1	0.0	3.1 (1)	0.0	7.7 (1)
f41.2	11.8 (2)	9.4 (3)	0.0	0.0
f42.2	0.0	3.1 (1)	0.0	0.0
f43.2	17.6 (3)	12.5 (4)	0.0	0.0
f60.9	0.0	3.1 (1)	28.6 (1)	7.7 (1)
g47	5.9 (1)	0.0	0.0	0.0

Abbreviations: F06.7 – Organic anxiety and depressive disorder; F10.2 – Alcohol dependence syndrome; F19.2 – Dependence syndrome due to use of other psychoactive substances; F32 – Depressive episode; F33 – Recurrent depressive disorder; F41.1 – Generalized anxiety disorder (GAD); F41.2 – Mixed anxiety and depressive disorder; F42.2 – Mixed obsessive–compulsive disorder; F43.2 – Adjustment disorders; F60.9 – Personality disorder, unspecified; G47 – Sleep disorders (insomnia, hypersomnia, parasomnias, circadian rhythm disturbances)

Table S14. Percentage and total number of Psychiatric Diagnoses by Group – Stage 2

Diagnosis	COVID-19 (n=17)	Control Group (n=32)	HCV (n=7)	TBEV (n=13)
d43	5.9 (1)	0.0	0.0	0.0
f10.2	0.0	3.1 (1)	0.0	0.0
f19.2	0.0	0.0	28.6 (2)	7.7 (1)
f32	11.8 (2)	6.2 (2)	14.3 (1)	15.4 (2)
f33	0.0	6.2 (2)	0.0	0.0
f41.1	0.0	3.1 (1)	0.0	15.4 (2)
f41.2	17.6 (3)	6.2 (2)	0.0	0.0
f42.2	0.0	3.1 (1)	0.0	0.0
f43.2	11.8 (2)	9.4 (3)	0.0	0.0
f60.9	0.0	3.1 (1)	28.6 (1)	7.7 (1)

Abbreviations: D43 – Neoplasm of uncertain or unknown behavior of brain and central nervous system; F10.2 – Alcohol dependence syndrome; F19.2 – Dependence syndrome due to use of other psychoactive substances; F32 – Depressive episode; F33 – Recurrent depressive disorder; F41.1 – Generalized anxiety disorder (GAD); F41.2 – Mixed anxiety and depressive disorder; F42.2 – Mixed obsessive–compulsive disorder; F43.2 – Adjustment disorders; F60.9 – Personality disorder, unspecified

Table S15. Comparison of patients differentiated by diagnosis in terms of changes in psychiatric diagnosis over the course of the study

Change in psychiatric diagnosis	Control group		Covid-19		HCV		TBEV		$\chi^2(9)$	*p*	Vc
	N	%	*N*	%	*N*	%	*N*	%			
No change	29	90,6%	8	47,1%	6	85,7%	11	84,6%	16,22	0,062	0,28
Diagnosis remission	2	6,3%	4	23,5%	0	0,0%	1	7,7%			
Diagnosis change	0	0,0%	2	11,8%	0	0,0%	0	0,0%			
Diagnosis onset	1	3,1%	3	17,6%	1	14,3%	1	7,7%			

Annotation. N – number of observations; χ^2 – chi-square test statistic; p – statistical significance; Vc – effect size indicator (Cramér's V).

Table S16. Comparison of patients differentiated by substance use in terms of changes in psychiatric diagnosis over the course of the study

Substance	Change in psychiatric diagnosis	Substance use: No		Substance use: Yes		$\chi^2(3)$	*p*	Vc
		N	%	*N*	%			
Cigarettes	No change	39	83,0%	15	68,2%	5,63	0,131	0,29
	Diagnosis remission	5	10,6%	2	9,1%			
	Diagnosis change	0	0,0%	2	9,1%			
	Diagnosis onset	3	6,4%	3	13,6%			
Alcohol	No change	47	81,0%	7	63,6%	5,95	0,114	0,29
	Diagnosis remission	6	10,3%	1	9,1%			
	Diagnosis change	2	3,4%	0	0,0%			
	Diagnosis onset	3	5,2%	3	27,3%			
Drugs	No change	51	79,7%	3	60,0%	7,01	0,072	0,32
	Diagnosis remission	7	10,9%	0	0,0%			
	Diagnosis change	2	3,1%	0	0,0%			

	Diagnosis onset	4	6,3%	2	40,0%

Annotation. N – number of observations; χ^2 – chi-square test statistic; p – statistical significance; Vc – effect size indicator (Cramér's V).

Table S17. Comparison of patients differentiated by substance use in terms of changes in psychiatric diagnosis throughout the study

		Comorbidities						
		No		Yes				
Disease		*N*	%	*N*	%	$\chi^2(3)$	*p*	Vc
Diabetes	No change	46	76,7%	8	88,9%	1,57	0,666	0,15
	Diagnosis remission	7	11,7%	0	0,0%			
	Diagnosis change	2	3,3%	0	0,0%			
	Diagnosis onset	5	8,3%	1	11,1%			
Hypertension	No change	35	79,5%	19	76,0%	1,79	0,618	0,16
	Diagnosis remission	4	9,1%	3	12,0%			
	Diagnosis change	2	4,5%	0	0,0%			
	Diagnosis onset	3	6,8%	3	12,0%			
Heart diseases	No change	49	80,3%	5	62,5%	2,67	0,446	0,20
	Diagnosis remission	5	8,2%	2	25,0%			
	Diagnosis change	2	3,3%	0	0,0%			
	Diagnosis onset	5	8,2%	1	12,5%			
Cancers	No change	52	82,5%	2	33,3%	10,86	**0,013**	0,40
	Diagnosis remission	6	9,5%	1	16,7%			
	Diagnosis change	1	1,6%	1	16,7%			
	Diagnosis onset	4	6,3%	2	33,3%			
Asthma/COPD	No change	52	78,8%	2	66,7%	10,67	**0,014**	0,39
	Diagnosis remission	7	10,6%	0	0,0%			
	Diagnosis change	1	1,5%	1	33,3%			
	Diagnosis onset	6	9,1%	0	0,0%			
Hyperlipidemia	No change	44	75,9%	10	90,9%	1,98	0,577	0,17
	Diagnosis remission	7	12,1%	0	0,0%			
	Diagnosis change	2	3,4%	0	0,0%			
	Diagnosis onset	5	8,6%	1	9,1%			

Annotation. N – number of observations; χ^2 – chi-square test statistic; p – statistical significance; Vc – effect size indicator (Cramér's V).

Table S18A. Post-hoc test results (Dunn's test with Bonferroni correction) for the comparison of patients differentiated by diagnosis in terms of IFN-γ, IL-1α, IL-4, IL-10, and MCP-1 levels.

	IFN- γ		IL-1α		IL-4		IL-10		MCP-1	
Group 1 - Group 2	*Z*	*p*	*Z*	*p*	*Z*	*p*	*Z*	*p*	*Z*	*p*
Control group-Covid-19	-3,78	**0,001**	-3,00	**0,016**	-2,74	**0,037**	-3,61	**0,002**	-2,94	**0,020**
Control group-TBEV	-3,71	**0,001**	-3,80	**0,001**	-3,57	**0,002**	-4,47	**<0,001**	-3,57	**0,002**
Control group-HCV	-3,32	**0,005**	-3,27	**0,007**	-3,16	**0,009**	-3,01	**0,016**	-3,31	**0,006**
Covid-19-TBEV	-0,23	1,000	-0,95	1,000	-0,96	1,000	-1,05	1,000	-0,79	1,000
Covid-19-HCV	-0,56	1,000	-1,03	1,000	-1,11	1,000	-0,38	1,000	-1,11	1,000

TBEV-HCV	0,36	1,000	0,24	1,000	0,31	1,000	0,46	1,000	0,44	1,000

Annotation. IFN-γ - interferon gamma; IL-1α - interleukin 1 alpha; IL-4 - interleukin 4; IL-10 - interleukin 10; MCP-1 - monocyte chemoattractant protein 1; p – adjusted p-value; Z - standardized test statistic from Dunn's post-hoc test

Table S18B. Post-hoc test results (Dunn's test with Bonferroni correction) for the comparison of patients differentiated by diagnosis in terms of IL-1ra and IL-6 levels

	IL-1ra		IL-6	
Group 1 - Group 2	*Z*	*p*	*Z*	*p*
Control group-Covid-19	-3,86	**0,001**	-4,27	**<0,001**
Control group-TBEV	-3,63	**0,002**	-3,31	**0,006**
Control group-HCV	-2,50	0,075	-1,94	0,313
Covid-19-TBEV	-0,10	1,000	-0,52	1,000
Covid-19-HCV	-0,26	1,000	-1,05	1,000
TBEV-HCV	0,33	1,000	0,60	1,000

Annotation. IL-1ra - interleukin 1 receptor antagonist; IL-6 - interleukin 6; p – adjusted p-value; Z - standardized test statistic from Dunn's post-hoc test

Table S18C. Post-hoc test results (Dunn's test with Bonferroni correction) for the comparison of patients differentiated by diagnosis in terms of IL-2Rα and IP-10 levels

	IL-2Rα		IP-10	
Group 1 - Group 2	*Z*	*p*	*Z*	*p*
Control group-Covid-19	-5,04	**<0,001**	-4,27	**<0,001**
Control group-TBEV	-1,95	0,309	-1,96	0,302
Control group-HCV	-2,88	**0,024**	-2,89	**0,024**
Covid-19-TBEV	-2,37	0,107	-1,73	0,499
Covid-19-HCV	-0,69	0,490	-0,17	1,000
TBEV-HCV	1,20	1,000	1,20	1,000

Annotation. IL-2Rα - interleukin 2 receptor alpha; IP-10- interferon gamma–induced protein 10; p – adjusted p-value; Z – standardized test statistic from Dunn's post-hoc test

Table S18D. Post-hoc test results (Dunn's test with Bonferroni correction) for the comparison of patients differentiated by diagnosis in terms of IL-12(p40), IL-17, and TNF-α levels

	IL-12(p40)		IL-17		TNF-α	
Group 1 - Group 2	*Z*	*p*	*Z*	*p*	*Z*	*p*
Control group-Covid-19	-2,52	0,070	-2,08	0,227	-2,36	0,109
Control group-TBEV	-4,60	**<0,001**	-3,42	**0,004**	-2,76	**0,035**
Control group-HCV	-2,81	**0,030**	-3,75	**<0,001**	-3,04	**0,014**
Covid-19-TBEV	-2,05	0,244	-1,36	1,000	-0,54	1,000
Covid-19-HCV	-0,92	1,000	-2,10	0,217	-1,24	1,000
TBEV-HCV	0,72	1,000	0,94	1,000	0,77	1,000

Annotation. IL-12(p40) - interleukin 12, p40 subunit; IL-17 - interleukin 17; p – adjusted p-value; TNF-α - tumor necrosis factor alpha; Z – standardized test statistic from Dunn's post-hoc test

Table S18E. Post-hoc test results (Dunn's test with Bonferroni correction) for the comparison of patients differentiated by diagnosis in terms of IL-8 and M-CSF levels

	IL-8		M-CSF	
Group 1 - Group 2	*Z*	*p*	*Z*	*p*
Control group-Covid-19	-3,39	**0,004**	-4,63	**<0,001**
Control group-TBEV	-0,84	1,000	-1,00	1,000
Control group-HCV	-2,18	0,178	-2,00	0,274
Covid-19-TBEV	-3,51	**0,003**	-2,88	**0,024**
Covid-19-HCV	-0,25	1,000	-1,24	1,000
TBEV-HCV	2,52	0,070	1,08	1,000

Annotation. IL-8 - interleukin 8; M-CSF - macrophage colony-stimulating factor; p – adjusted p-value; Z – standardized test statistic from Dunn's post-hoc test

Table S18F. Post-hoc test results (Dunn's test with Bonferroni correction) for the comparison of patients differentiated by diagnosis in terms of RANTES levels.

	RANTES	
Group 1 - Group 2	*Z*	*p*
Control group-Covid-19	-1,21	1,000
Control group-TBEV	-3,72	**0,001**
Control group-HCV	-2,03	0,254
Covid-19-TBEV	-2,33	0,118
Covid-19-HCV	-1,08	1,000
TBEV-HCV	0,80	1,000

Annotation. p – adjusted p-value; RANTES - regulated upon activation, normal T cell expressed and secreted; Z – standardized test statistic from Dunn's post-hoc test

Table S19. Hierarchical logistic regression models explaining the occurrence of psychiatric diagnosis at Stage 1 and Stage 2 of the study based on selected serum parameters

							95% *CI*	
Dependant variable		*B*	*SE*	*Wald*	*p*	*Exp*(*B*)	*LL*	*UL*
Psychiatric diagnosis – stage 1 (0 – no; 1 – yes)	**$\chi^2(9) = 34{,}01$; $p < 0{,}001$; R^2 Nagelkerke = 0,523**							
	IFN-α2	-1,22	0,59	4,32	**0,038**	0,30	0,09	0,93
	IL-2	0,60	0,38	2,43	0,119	1,82	0,86	3,85
	IL-2Rα	0,06	0,02	6,54	**0,011**	1,06	1,01	1,10
	IL-9	0,01	0,00	3,17	0,075	1,01	1,00	1,02
	IL-10	0,10	0,07	1,98	0,160	1,10	0,96	1,26
	IL-12 (p40)	0,05	0,03	2,54	0,111	1,05	0,99	1,13
	IL-17	-0,24	0,10	5,74	**0,017**	0,79	0,64	0,96
	IP-10	0,00	0,00	6,87	**0,009**	0,99	0,99	1,00
	TNF-α	-0,01	0,01	1,30	0,254	0,99	0,96	1,01
	Constant	-4,14	2,93	2,00	0,158	0,02		
	**$\chi^2(3) = 8{,}24$; $p = 0{,}041$; R^2 Nagelkerke = 0,153**							

Psychiatric diagnosis – stage 2 (0 – no; 1 – yes)	IL-1β	0,20	0,12	2,66	0,103	1,22	0,96	1,56
	IL-17	-0,13	0,08	2,56	0,110	0,88	0,75	1,03
	RANTES	0,00	0,00	4,20	**0,040**	1,01	1,00	1,01
	Constant	-2,21	0,99	4,98	**0,026**	0,11		

Annotation. B – unstandardized regression coefficient; SE – standard error; Exp(B) – exponentiated coefficient

Table S20. Hierarchical logistic regression models explaining the occurrence of specific psychiatric symptoms at Stage 1 of the study based on selected serum parameters

							95% *CI*	
Dependant variable		*B*	*SE*	*Wald*	*p*	*Exp*(*B*)	*LL*	*UL*
Anxiety (HADS) (0 – no; 1 – yes)	**$\chi^2(5) = 10{,}94$; $p = 0{,}053$; R^2 Nagelkerke = 0,222**							
	IL-12 (p70)	0,06	0,03	3,51	0,061	1,06	1,00	1,13
	IL-12 (p40)	0,05	0,02	6,00	**0,014**	1,05	1,01	1,09
	IL-17	-0,11	0,08	2,04	0,153	0,90	0,77	1,04
	RANTES	<0,01	<0,01	3,10	0,078	1,00	1,00	1,00
	TNF-β	0,01	0,01	3,01	0,083	1,01	1,00	1,02
	Constant	-2,63	2,25	1,37	0,241	0,07		
Depression (HADS) (0 – no; 1 – yes)	**$\chi^2(9) = 31{,}28$; $p < 0{,}001$; R^2 Nagelkerke = 0,534**							
	Eotaxin	-0,02	0,01	2,90	0,089	0,98	0,96	1,00
	IL-2Rα	0,04	0,02	3,67	0,056	1,04	1,00	1,09
	IL-7	0,11	0,05	4,73	**0,030**	1,12	1,01	1,24
	IL-8	-0,38	0,24	2,47	0,116	0,68	0,42	1,10
	IL-9	0,01	0,01	5,74	**0,017**	1,01	1,00	1,02
	IL-10	0,11	0,06	3,39	0,066	1,11	0,99	1,25
	IL-17	-0,21	0,15	1,85	0,174	0,81	0,60	1,10
	IP-10	<0,01	<0,01	4,05	**0,044**	1,00	0,99	1,00
	RANTES	<0,01	<0,01	6,12	**0,013**	1,00	0,99	1,00
	Constant	-0,62	2,06	0,09	0,763	0,54		
Sleep disorders (0 – no; 1 – yes)	**$\chi^2(4) = 12{,}29$; $p = 0{,}015$; R^2 Nagelkerke = 0,251**							
	IL-9	0,01	<0,01	2,36	0,125	1,01	1,00	1,01
	IL-12 (p70)	0,11	0,09	1,52	0,218	1,11	0,94	1,31
	IP-10	<0,01	<0,01	2,51	0,113	1,00	0,99	1,00
	RANTES	<0,01	<0,01	3,18	0,075	1,00	1,00	1,00
	Constant	-1,52	1,88	0,65	0,418	0,22		
Cognitive impairment (0 – no; 1 – yes)	**$\chi^2(7) = 25{,}49$; $p < 0{,}001$; R^2 Nagelkerke = 0,573**							
	Eotaxin	0,05	0,02	4,87	**0,027**	1,05	1,01	1,10
	IL-2Rα	0,05	0,03	3,93	**0,047**	1,05	1,00	1,11
	IL-12 (p40)	-0,16	0,11	2,18	0,140	0,86	0,70	1,05
	IL-17	-1,43	0,60	5,72	**0,017**	0,24	0,07	0,77
	RANTES	<0,01	<0,01	5,86	**0,016**	1,00	1,00	1,00
	SCGF-β	<0,01	<0,01	3,29	0,070	1,00	1,00	1,00

Dependant variable		B	SE	Wald	p	Exp(B)	95% CI LL	95% CI UL
	TNF-β	-0,01	0,01	2,23	0,136	0,99	0,97	1,00
	Constant	8,92	5,84	2,33	0,126	7458,07		
Loss of energy (0 – no; 1 – yes)	**$\chi^2(4) = 17,26$; $p = 0,002$; R^2 Nagelkerke = 0,367**							
	IL-8	-0,31	0,17	3,22	0,073	0,73	0,52	1,03
	IL-9	0,01	<0,01	3,50	0,061	1,01	1,00	1,02
	IL-10	0,12	0,04	7,05	**0,008**	1,12	1,03	1,22
	RANTES	<0,01	<0,01	5,73	**0,017**	0,99	0,99	1,00
	Constant	-1,37	1,92	0,51	0,476	0,25		
Depressed mood (0 –no; 1 – yes)	**$\chi^2(7) = 21,70$; $p = 0,003$; R^2 Nagelkerke = 0,385**							
	IL-2Rα	0,02	0,01	3,03	0,082	1,02	1,00	1,05
	IL-8	-0,17	0,12	2,01	0,156	0,84	0,67	1,07
	IL-9	0,01	<0,01	3,18	0,075	1,01	1,00	1,02
	IL-10	0,08	0,05	2,67	0,102	1,09	0,98	1,20
	IL-12 (p40)	0,04	0,02	3,51	0,061	1,04	1,00	1,08
	IP-10	<0,01	<0,01	4,20	**0,040**	0,99	0,99	1,00
	RANTES	<0,01	<0,01	6,04	**0,014**	1,00	0,99	1,00
	Constant	-1,41	1,75	0,66	0,418	0,24		
Anxiety (0 – no; 1 – yes)	**$\chi^2(3) = 9,24$; $p = 0,026$; R^2 Nagelkerke = 0,179**							
	IL-12 (p70)	0,05	0,03	2,02	0,155	1,05	0,98	1,12
	IL-12 (p40)	0,03	0,02	4,71	**0,030**	1,03	1,00	1,07
	IL-17	-0,17	0,08	4,45	**0,035**	0,84	0,72	0,99
	Constant	0,48	0,87	0,30	0,584	1,61		

Annotation. B – unstandardized regression coefficient; SE – standard error; Exp(B) – exponentiated coefficient

Table S21. Hierarchical logistic regression models explaining the occurrence of specific psychiatric symptoms at Stage 2 of the study based on selected serum parameters.

Dependent variable		B	SE	Wald	p	Exp(B)	95% CI LL	95% CI UL
Anxiety (HADS) (0 – no; 1 – yes)	**$\chi^2(2) = 4,87$; $p = 0,088$; R^2 Nagelkerke = 0,117**							
	IL-6	0,13	0,07	3,61	0,058	1,14	1,00	1,30
	IL-12 (p70)	-0,18	0,12	2,21	0,137	0,84	0,67	1,06
	Constant	-1,78	0,46	15,28	**<0,001**	0,17		
Depression (HADS) (0 – no; 1 – yes)	**$\chi^2(6) = 28,44$; $p < 0,001$; R^2 Nagelkerke = 0,627**							
	IL-2	-1,76	0,89	3,94	**0,047**	0,17	0,03	0,98
	IL-12 (p40)	0,12	0,05	4,82	**0,028**	1,12	1,01	1,25
	IL-17	-0,48	0,27	3,21	0,073	0,62	0,36	1,05
	MIP-1α	1,08	0,81	1,77	0,184	2,95	0,60	14,54
	SCGF-β	<0,01	<0,01	6,48	**0,011**	1,00	1,00	1,00
	TNF-α	-0,09	0,06	2,61	0,107	0,91	0,82	1,02

Dependent variable		*B*	*SE*	*Wald*	*p*	*Exp*(*B*)	95% *CI* *LL*	*UL*
	Constant	5,89	5,27	1,25	0,264	359,68		
Sleep disorders (0 – no; 1 – yes)	**χ^2(2) = 7,53; *p* = 0,023; R^2 Nagelkerke = 0,154**							
	IL-7	0,04	0,02	3,48	0,062	1,04	1,00	1,09
	IL-10	-0,12	0,06	4,20	**0,040**	0,89	0,79	0,99
	Constant	-1,58	0,75	4,43	**0,035**	0,21		
Cognitive impairment (0 – no; 1 – yes)	**χ^2(11) = 27,80; *p* = 0,003; R^2 Nagelkerke = 0,493**							
	Eotaxin	-0,02	0,01	3,49	0,062	0,98	0,97	1,00
	IL-1β	0,68	0,34	4,03	**0,045**	1,98	1,02	3,87
	IL-2Rα	0,05	0,02	7,16	**0,007**	1,05	1,01	1,09
	IL-6	-0,18	0,18	1,01	0,314	0,84	0,59	1,18
	IL-7	0,06	0,04	2,65	0,103	1,07	0,99	1,15
	IL-8	0,08	0,04	3,98	**0,046**	1,08	1,00	1,17
	IL-12 (p40)	-0,06	0,04	1,95	0,163	0,94	0,86	1,02
	IP-10	<0,01	<0,01	2,56	0,109	1,00	1,00	1,00
	MIP-1α	-1,36	0,67	4,11	**0,043**	0,26	0,07	0,96
	RANTES	<0,01	<0,01	10,26	**0,001**	1,00	1,00	1,00
	TNF-α	-0,10	0,04	6,25	**0,012**	0,90	0,83	0,98
	Constant	-2,95	2,35	1,58	0,209	0,05		
Loss of energy (0 – no; 1 – yes)	**χ^2(1) = 2,71; *p* = 0,100; R^2 Nagelkerke = 0,068**							
	IL-10	-0,08	0,06	1,76	0,184	0,93	0,82	1,04
	Constant	-1,10	0,54	4,24	**0,039**	0,33		
Depressed mood (0 –no; 1 – yes)	**χ^2(2) = 8,12; *p* = 0,017; R^2 Nagelkerke = 0,179**							
	IL-12 (p70)	-0,43	0,24	3,31	0,069	0,65	0,41	1,03
	IL-12 (p40)	0,04	0,02	5,41	**0,020**	1,04	1,01	1,07
	Constant	-1,24	0,47	7,01	**0,008**	0,29		
Anxiety (0 – no; 1 – yes)	**χ^2(3) = 14,33; *p* = 0,002; R^2 Nagelkerke = 0,271**							
	IL-1β	0,31	0,17	3,45	0,063	1,36	0,98	1,88
	IL-10	-0,14	0,07	3,82	0,051	0,87	0,75	1,00
	RANTES	<0,01	<0,01	5,98	**0,014**	1,00	1,00	1,00
	Constant	-5,53	1,78	9,66	**0,002**	<0,01		

Annotation. B – unstandardized regression coefficient; SE – standard error; Exp(B) – exponentiated coefficient

Table S22. Hierarchical linear regression models explaining anxiety and depression levels at Stage 1 of the study based on selected serum parameters.

Dependent variable		*B*	*SE*	*Beta*	*t*	*p*	95% *CI* *LL*	*UL*
Anxiety (HADS)	***F*(1; 68) = 10,94; *p* = 0,038; $R^2_{adj.}$ = 0,049**							
	IL-1β	0,36	0,17	0,25	2,12	**0,038**	0,02	0,69
	Constant	2,98	1,29		2,13	**0,024**	0,41	5,56

Dependent variable		B	SE	Beta	t	p	95% CI LL	95% CI UL
Depression (HADS)	**$F(3; 68) = 5{,}12$; $p = 0{,}003$; $R^2_{adj.} = 0{,}154$**							
	IL-8	-0,04	0,02	-0,23	-1,98	0,052	-0,08	<0,01
	IL-10	0,16	0,05	0,38	3,13	**0,003**	0,06	0,26
	RANTES	<0,01	<0,01	-0,30	-2,55	**0,013**	-0,01	0,00
	Constant	7,51	1,52		4,96	**<0,001**	4,49	10,54

Annotation. B – unstandardized regression coefficient; SE – standard error; Beta – standardized regression coefficient.

Table S23. Hierarchical linear regression models explaining anxiety and depression levels at Stage 2 of the study based on selected serum parameters

Dependent variable		B	SE	Beta	t	p	95% CI LL	95% CI UL
Anxiety (HADS)	**$F(1; 68) = 2{,}39$; $p = 0{,}127$; $R^2_{adj.} = 0{,}020$**							
	IL-2Rα	0,01	0,01	0,19	1,55	0,127	-0,02	0,03
	Constant	3,91	0,70		5,62	**<0,001**	2,52	5,31
Depression (HADS)	**$F(3; 68) = 10{,}95$; $p < 0{,}001$; $R^2_{adj.} = 0{,}305$**							
	IP-10	<0,01	<0,01	0,23	2,00	**0,050**	<0,01	0,01
	RANTES	<0,01	<0,01	-0,19	-1,87	0,066	<0,01	<0,01
	SCGF-β	<0,01	<0,01	0,42	3,67	**<0,001**	<0,01	<0,01
	Constant	-0,11	1,52		-0,07	0,942	-3,15	2,93

Annotation. B – unstandardized regression coefficient; SE – standard error; Beta – standardized regression coefficient.

Table S24. Comparison of psychiatric diagnoses between patients stratified by high and low serum cytokine levels based on cluster analysis

		Group 1 – high cytokine levels		Group 2 – low cytokine levels				
		N	%	*N*	%	$\chi^2(3)$	*p*	Vc
Diagnosis	Control group	1	7,1%	31	56,4%	11,15	**0,011**	0,40
	Covid-19	6	42,9%	11	20,0%			
	HCV	2	14,3%	5	9,1%			
	TBEV	5	35,7%	8	14,5%			

Annotation. N – number of observations; χ^2 – chi-square test statistic; p – statistical significance; Vc – effect size indicator (Cramér's V).

Table S25. Comparison of psychiatric symptom presence at Stage 1 and Stage 2 between patients stratified by plasma parameter profiles based on cluster analysis

Variable		Group 1 – high plasma parameter values		Group 2 – moderately low plasma parameter values				
		N	%	*N*	%	χ^2	*p*	ϕ
Sleep disorders - stage 1	No	12	85,7%	42	76,4%	0,57	0,718	0,09
	Yes	2	14,3%	13	23,6%			
Cognitive impairment - stage 1	No	13	92,9%	47	85,5%	0,54	0,674	0,09
	Yes	1	7,1%	8	14,5%			
Loss of energy - stage 1	No	13	92,9%	44	80,0%	1,28	0,436	0,14
	Yes	1	7,1%	11	20,0%			
Anxiety - stage 1	No	11	78,6%	42	76,4%	0,03	1,000	0,02
	Yes	3	21,4%	13	23,6%			
Depression - stage 1	No	13	92,9%	38	69,1%	3,27	0,094	0,22
	Yes	1	7,1%	17	30,9%			
Sleep disorders - stage 2	No	12	85,7%	40	72,7%	1,01	0,491	0,12
	Yes	2	14,3%	15	27,3%			
Cognitive impairment - stage 2	No	10	71,4%	42	76,4%	0,15	0,734	0,05
	Yes	4	28,6%	13	23,6%			
Loss of energy - stage 2	No	13	92,9%	46	83,6%	0,77	0,674	0,11
	Yes	1	7,1%	9	16,4%			
Anxiety - stage 2	No	11	78,6%	47	85,5%	0,40	0,683	0,08
	Yes	3	21,4%	8	14,5%			
Depression - stage 2	No	12	85,7%	48	87,3%	0,02	1,000	0,02
	Yes	2	14,3%	7	12,7%			

Annotation. N – number of observations; χ^2 – chi-square test statistic; p – statistical significance; ϕ – effect size indicator

Table S26. Comparison of anxiety and depression severity assessed by the HADS at Stage 1 and Stage 2 between patients stratified by plasma parameter profiles based on cluster analysis

Dependent variable	Group 1 – high plasma parameter values (*n* = 14)			Group 2 – moderately low plasma parameter values (*n* = 55)					
	Mean rank	*M*	*SD*	Mean rank	*M*	*SD*	*Z*	*p*	η^2
Anxiety - stage 1	39,46	6,07	3,58	33,86	5,40	4,06	-0,94	0,348	0,01
Depression – stage 1	31,43	3,50	3,90	35,91	5,24	5,00	-0,75	0,452	<0,01
Anxiety - stage 2	39,29	5,21	3,17	33,91	4,73	3,12	-0,90	0,367	0,01
Depression - stage 2	42,75	4,86	4,52	33,03	3,27	3,61	-1,63	0,102	0,04

Annotation. n – number of observations; M – mean; SD – standard deviation; Z – test statistic value; p – statistical significance; η^2 – effect size indicator

Table S27. Comparison of patients differentiated by history of COVID-19 infection in terms of changes in psychiatric diagnosis over the course of the study

Change in psychiatric diagnosis	History of Covid-19				$\chi^2(3)$	*p*	Vc
	No		Yes				
	N	%	*N*	%			
No change	29	74,4%	25	83,3%	1,97	0,580	0,17
Diagnosis remission	4	10,3%	3	10,0%			
Diagnosis change	2	5,1%	0	0,0%			
Diagnosis onset	4	10,3%	2	6,7%			

Annotation. N – number of observations; χ^2 – chi-square test statistic; p – statistical significance; Vc – effect size indicator (Cramér's V).

Table S28. Comparison of patients differentiated by history of COVID-19 infection in terms of the presence of specific psychiatric symptoms at Stage 1 of the study

Psychiatric symptoms at Stage 1		History of Covid-19				$\chi^2(1)$	*p*	ϕ
		No		Yes				
		N	%	*N*	%			
Anxiety- HADS	No	31	79,5%	22	73,3%	0,36	0,577	0,07
	Yes	8	20,5%	8	26,7%			
Depression - HADS	No	32	82,1%	19	63,3%	3,08	0,101	0,21
	Yes	7	17,9%	11	36,7%			
Sleep disorders	No	28	71,8%	26	86,7%	2,20	0,156	0,18
	Yes	11	28,2%	4	13,3%			
Cognitive impairment	No	33	84,6%	27	90,0%	0,43	0,722	0,08
	Yes	6	15,4%	3	10,0%			
Loss of energy	No	33	84,6%	24	80,0%	0,25	0,751	0,06
	Yes	6	15,4%	6	20,0%			
Depressed mood	No	30	76,9%	19	63,3%	1,52	0,286	0,15
	Yes	9	23,1%	11	36,7%			
Anxiety	No	29	74,4%	20	66,7%	0,49	0,595	0,08
	Yes	10	25,6%	10	33,3%			
Obsessions/compulsions	No	37	94,9%	30	100,0%	1,58	0,501	0,15
	Yes	2	5,1%	0	0,0%			

Annotation. N – number of observations; χ^2 – chi-square test statistic; p – statistical significance; ϕ – effect size indicator

Table S29. Comparison of patients differentiated by history of COVID-19 infection in terms of the presence of specific psychiatric symptoms at Stage 2 of the study.

Psychiatric symptoms at Stage 2		History of Covid-19				$\chi^2(1)$	*p*	ϕ
		No		Yes				
		N	%	*N*	%			
Anxiety- HADS	No	35	89,7%	23	76,7%	2,16	0,190	0,18
	Yes	4	10,3%	7	23,3%			
Depression - HADS	No	33	84,6%	27	90,0%	0,43	0,722	0,08
	Yes	6	15,4%	3	10,0%			

Sleep disorders	No	30	76,9%	22	73,3%	0,12	0,783	0,04
	Yes	9	23,1%	8	26,7%			
Cognitive impairment	No	32	82,1%	20	66,7%	2,16	0,167	0,18
	Yes	7	17,9%	10	33,3%			
Loss of energy	No	34	87,2%	25	83,3%	0,20	0,737	0,05
	Yes	5	12,8%	5	16,7%			
Depressed mood	No	31	79,5%	25	83,3%	0,16	0,764	0,05
	Yes	8	20,5%	5	16,7%			
Anxiety	No	29	74,4%	21	70,0%	0,16	0,788	0,05
	Yes	10	25,6%	9	30,0%			
Obsessions/compulsions	No	38	97,4%	30	100,0%	0,78	1,000	0,11
	Yes	1	2,6%	0	0,0%			

Annotation. N – number of observations; χ^2 – chi-square test statistic; p – statistical significance; ϕ – effect size indicator

Table S30. Comparison of patients differentiated by history of COVID-19 infection in terms of changes in the presence of specific psychiatric symptoms over the course of the study

		History of Covid-19						
		No		Yes				
Change over the course of the study		*N*	%	*N*	%	$\chi^2(2)$	*p*	Vc
Anxiety- HADS	No change	33	84,6%	23	76,7%	1,75	0,416	0,16
	Symptom remission	5	12,8%	4	13,3%			
	Symptom onset	1	2,6%	3	10,0%			
Depression - HADS	No change	34	87,2%	22	73,3%	5,77	0,056	0,29
	Symptom remission	3	7,7%	8	26,7%			
	Symptom onset	2	5,1%	0	0,0%			
Sleep disorders	No change	33	84,6%	26	86,7%	4,40	0,111	0,25
	Symptom remission	4	10,3%	0	0,0%			
	Symptom onset	2	5,1%	4	13,3%			
Cognitive impairment	No change	32	82,1%	23	76,7%	4,19	0,123	0,25
	Symptom remission	3	7,7%	0	0,0%			
	Symptom onset	4	10,3%	7	23,3%			
Loss of energy	No change	36	92,3%	25	83,3%	1,37	0,505	0,14
	Symptom remission	2	5,1%	3	10,0%			
	Symptom onset	1	2,6%	2	6,7%			
Depressed mood	No change	32	82,1%	22	73,3%	2,54	0,281	0,19
	Symptom remission	4	10,3%	7	23,3%			
	Symptom onset	3	7,7%	1	3,3%			
Anxiety	No change	33	84,6%	23	76,7%	0,77	0,681	0,11
	Symptom remission	3	7,7%	4	13,3%			
	Symptom onset	3	7,7%	3	10,0%			
Obsessions/compulsions	No change	38	97,4%	30	100,0%	0,78	1,000	0,11
	Symptom remission	1	2,6%	0	0,0%			
	Symptom onset	0	0,0%	0	0,0%			

Annotation. N – number of observations; χ^2 – chi-square test statistic; p – statistical significance; Vc – effect size indicator (Cramér's V).

Table S31. Comparison of patients differentiated by history of COVID-19 infection in terms of anxiety and depression severity at Stage 1 of the study

	History of Covid-19									
	No (*n* = 39)		Yes (*n* = 30)					95% *CI*		
1 stage	*M*	*SD*	*M*	*SD*	*t*	*df*	*p*	*LL*	*UL*	*d* Cohen
Anxiety	5,15	3,93	6,03	3,99	-0,92	67	0,363	-2,80	1,04	0,22
Depression	4,36	4,28	5,57	5,44	-1,00[a]	53,91	0,321	-3,63	1,21	0,25

Annotation. n – number of observations; M – mean; SD – standard deviation; t – test statistic value; df – degrees of freedom; p – statistical significance; CI – confidence interval for the difference between means; LL and UL – lower and upper limits of the confidence interval.

[a] Levene's test was statistically significant – the Welch correction was applied.

Table S32. Comparison of patients differentiated by history of COVID-19 infection in terms of anxiety and depression severity at Stage 2 of the study

	History of Covid-19									
	No (*n* = 39)		Yes (*n* = 30)					95% *CI*		
2 stage	*M*	*SD*	*M*	*SD*	*t*	*df*	*p*	*LL*	*UL*	*d* Cohen
Anxiety	4,90	3,28	4,73	2,94	0,22	67	0,830	-1,35	1,68	0,05
Depression	4,03	4,20	3,03	3,29	1,07	67	0,290	-0,86	2,85	0,26

Annotation. n – number of observations; M – mean; SD – standard deviation; t – test statistic value; df – degrees of freedom; p – statistical significance; CI – confidence interval for the difference between means; LL and UL – lower and upper limits of the confidence interval.

Table S33. Comparison of patients differentiated by history of COVID-19 infection in terms of changes in anxiety and depression severity between Stage 1 and Stage 2 of the study

	History of Covid-19									
	No (*n* = 39)		Yes (*n* = 30)					95% *CI*		
Change over the course of the study	*M*	*SD*	*M*	*SD*	*t*	*df*	*p*	*LL*	*UL*	*d* Cohen
Anxiety	-0,26	2,44	-1,30	3,63	1,36[a]	48,15	0,181	-0,50	2,59	0,35
Depression	-0,33	3,72	-2,53	4,11	2,33	67	**0,023**	0,31	4,09	0,56

Annotation. n – number of observations; M – mean; SD – standard deviation; t – test statistic value; df – degrees of freedom; p – statistical significance; CI – confidence interval for the difference between means; LL and UL – lower and upper limits of the confidence interval.

[a] Levene's test was statistically significant – the Welch correction was applied.
